# Solvent-based post-processing to fill layer gaps in FFF-printed polypropylene fluidic chips and their application to flow injection analysis

**DOI:** 10.1007/s44211-026-00954-6

**Published:** 2026-08-03

**Authors:** Tomohisa Yamashita

**Affiliations:** Toyama Institute of Health, 17-1 Nakataikoyama, Imizu, Toyama 939-0363 Japan

**Keywords:** Polypropylene, Fluidic chip, 3D print, Fused filament fabrication, Flow injection

## Abstract

The development of new analytical tools remains a powerful approach in analytical chemistry, and three-dimensional (3D) printing has gained increasing popularity as a manufacturing technique in recent years. The fabrication of experimental tools using 3D printers has attracted attention in many fields. However, objects fabricated by fused filament fabrication (FFF) may contain gaps between printed paths, and which can cause leakage when liquid is introduced. In this study, solvent-based post-processing was performed in order to fill layer gaps in FFF-printed polypropylene fluidic chips. As methods, a compression process and solvent-based post-processing were applied. In the compression process, a polypropylene fluidic chip was compressed using a compression tool while being heated. After this compression process, solvent-based post-processing was conducted. The post-processed chip was used to measure peak profile during flow-injection measurement. As a result, peak profiles were obtained, and no leakage from the chip was observed during the measurements.

**Graphical abstract:**

**Figure:**
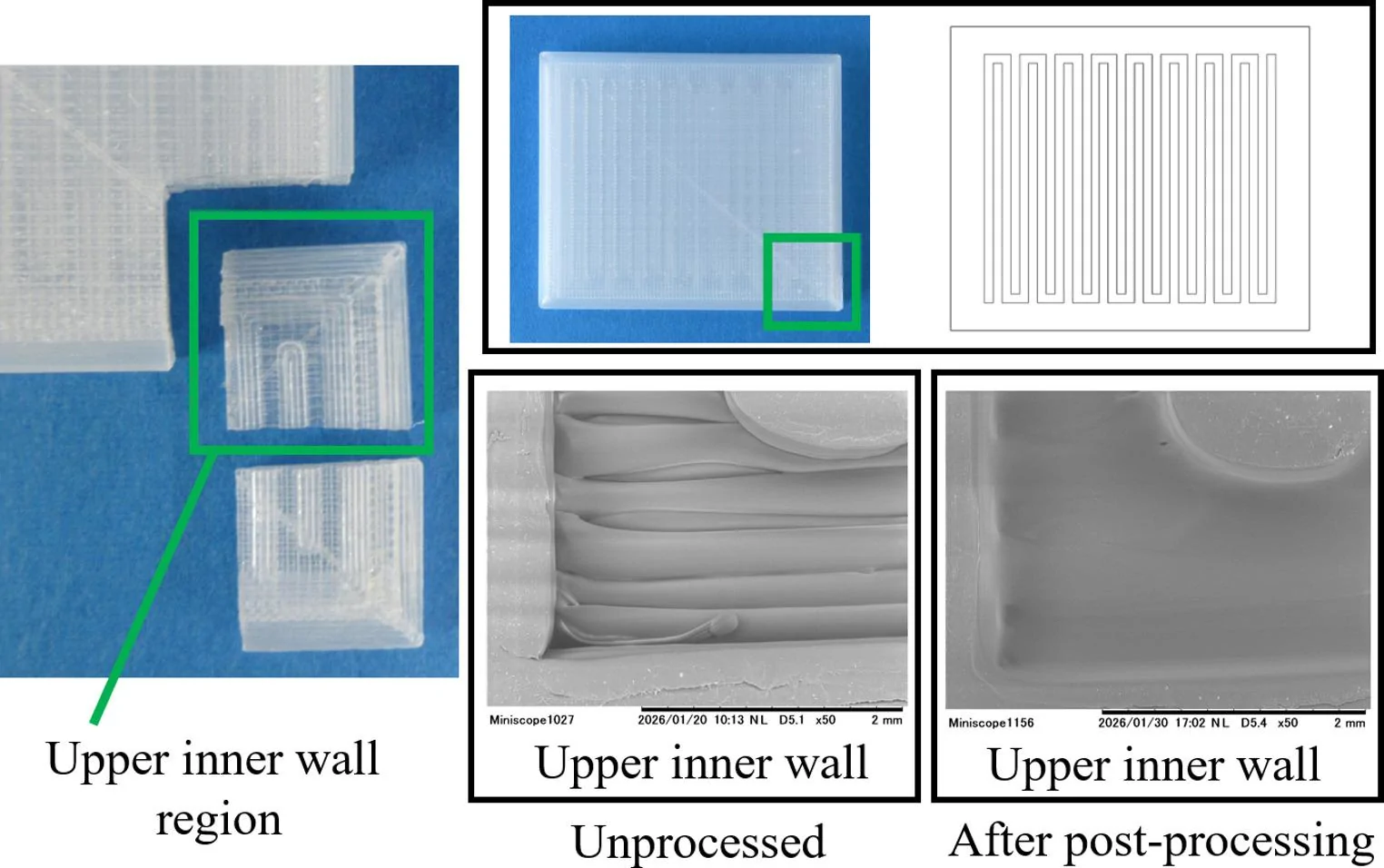

**Supplementary Information:**

The online version contains supplementary material available at https://doi.org/10.1007/s44211-026-00954-6.

## Introduction

In analytical chemistry, the development of new analytical tools is a powerful approach, and in recent years manufacturing using three-dimensional (3D) printing has gained increasing popularity. The fabrication of experimental tools using 3D printers has attracted attention in many fields [1–10]. The advantage of 3D printing is that it enables the inexpensive production of objects with complex shapes designed on a computer. Therefore, by using a 3D printer, researchers can fabricate their own new experimental tools.

Among the materials used for 3D printing, polypropylene (PP) is an attractive option. PP is widely used for laboratory equipment in many fields because of its high resistance to acids and bases, and it is also inexpensive compared with many other engineering plastics. Hence, it is advantageous to fabricate custom-shaped laboratory tools using PP. In recent years, various attempts have been reported to fabricate PP experimental tools using 3D printing [11–17]. However, the number of reported studies remains limited.

Generally, Selective Laser Sintering (SLS) and Fused Filament Fabrication (FFF) are mainly used for 3D printing of PP objects. When SLS is used to form PP objects, small PP particles are melted by a laser to form the object. In this process, unmelted particles remain inside the object; therefore, they must be removed from after printing. However, for objects with narrow and complex hollow internal structures, such as fluidic chips, removing these particles is difficult. Furthermore, SLS 3D printers are typically large and expensive.

In contrast, in FFF, small overhangs can be printed without support materials. Therefore, fluidic chips with small channel widths can be fabricated with hollow internal structures. In addition, FFF is inexpensive and commercially available for home use. For this reason, FFF is considered more advantageous than SLS for fabricating PP fluidic chips. (Although the term Fused Deposition Modeling (FDM) also used, FDM and FFF refer to essentially the same technology. FDM is a trademark, whereas FFF is the generic term.)

However, objects fabricated by FFF may contain gaps between printed paths, and which can cause leakage when liquid is introduced [2]. In addition, once liquid enters these gaps, it may become trapped and difficult to remove. In analytical experiments, this can lead to contamination or carryover. These gaps have long been a challenge when such objects are used as liquid containers. One effective approach to address this issue is solvent-based post-processing. In this process, the surface polymer is partially dissolved using a solvent, allowing the gaps to be filled with the polymer. As a result, leakage reduction is expected.

For materials other than polypropylene, several studies have reported methods for smoothing layer lines by partially dissolving the polymer surface through solvent-based post-processing [19–23]. Among these, Reference 19 reported post-processing aimed at preventing leakage.

For polypropylene, reports on solvent-based post-processing remain scarce. If a method to fill layer gaps in FFF‑printed PP objects using solvent-based post-processing can be established, it would be highly useful.

However, because solvent-based post-processing slightly alters surface features, a method that enables selective treatment of specific areas is desirable to avoid excessive modification. For example, external post-processing is sufficient to prevent leakage in laboratory vessels such as beakers. However, for analytical tools such as fluidic chips—where liquids typically flow through internal channels—treating only the external surface is not effective for leak reduction. Therefore, a method that enables selective post-processing of internal surfaces is preferable.

To the best of the author’s knowledge, only limited reports have addressed solvent-based post-processing of FFF-printed polypropylene parts, and these have primarily focused on external surfaces[24]. Furthermore, the author could not identify previous reports describing solvent-based post-processing selectively applied only to internal surfaces of FFF-printed PP structures in either the academic or patent literature.

If a reliable method for fabricating leakage-free polypropylene (PP) fluidic chips can be established, such chips could be applied to analytical chemistry, for example in flow-injection analysis (FIA). One advantage of performing FIA on a fluidic chip is the ability to make the flow‑path layout more compact. In conventional FIA systems, the flow path is constructed from tubing, and the allowable curvature is limited, which restricts the degree of miniaturization and densification. In contrast, fluidic chips can accommodate channels with higher curvature, enabling more complex and densely packed flow-path designs. Furthermore, the use of PP as the chip material broadens the range of solvents that can be employed.

In this study, solvent-based post-processing was performed in order to fill layer gaps in FFF-printed polypropylene fluidic chips. As methods, a thermal binding process and solvent-based post-processing were applied. Subsequently, flow-injection measurements were conducted using the fluidic chip.

## Experimental

### Reagents and chemicals

Heptane (Cat. No. 085–00151), ethanol (Cat. No. 052-03343) and methyl orange (Cat. No. 131–02862) were obtained from FUJIFILM Wako Pure Chemical Industries (Osaka, Japan). Allura red AC (Cat. No. A0943) was obtained from Tokyo Chemical Industry Co., Ltd. (Tokyo, Japan). PP filament (HEP-3DF) was purchased from Hokuriku Engineer Plastic Co., Ltd. (Imizu, Toyama, Japan). Water was purified using a Milli-Q system (Millipore) and used in the measurement experiments.

### Apparatus

A 3D printer (Lepton 2, Magnarecta, Japan) was used. A water bath (pair stirrer PS-100, EYELA) was used. An air pump (SSPP-2 S, Suisaku Corporation) was used to supply air. A drilling machine (No.28128, Proxxon) was used to drill holes in aluminum materials. The surface structure of samples was observed using a MiniScope TM3000 (Hitachi, Tokyo, Japan).

### Making of compression tool

The components of the compression tool are shown in Fig. S1(Supporting Information). Aluminum plates and aluminum angles were processed using a saw and a drilling machine to fabricate the compression tool components. Type TY springs (Tokyo Hatsujyo Manufacturing Co., Ltd.) were used as compression springs. Stainless steel bolts, nuts and washers were purchased and used.

### 3D printing of polypropylene chip

Blender (open-source 3D computer graphics software) was used to design the 3D model of the fluidic chip. The 3D model data of were exported as a standard triangulated language (STL) file. The 3D models were printed using a 3D printer (Lepton 2, Magnarecta, Hyogo, Japan). MatterControl 1.6 was used as the 3D printing software, and MatterSlice was used as the slicer. The 3D printing parameters are shown in Fig. S2 (Supporting Information). Before printing, Ultra Strong Double-Sided Adhesive Tape for PE / PP (No. 5015, Nitto) was applied to the print bed, and a PP sheet (thickness: 0.2 mm) was placed on the tape. PP filament was used to fabricate the fluidic chip. During printing, extrusion rate was set to 110% of the standard value. After printing, the fluidic chip was removed from the PP sheet. If the PP sheet adhered strongly and could not be removed, it was cut along the edges of the fluidic chip.

### Compression of fluidic chip

**Fig. 1:**
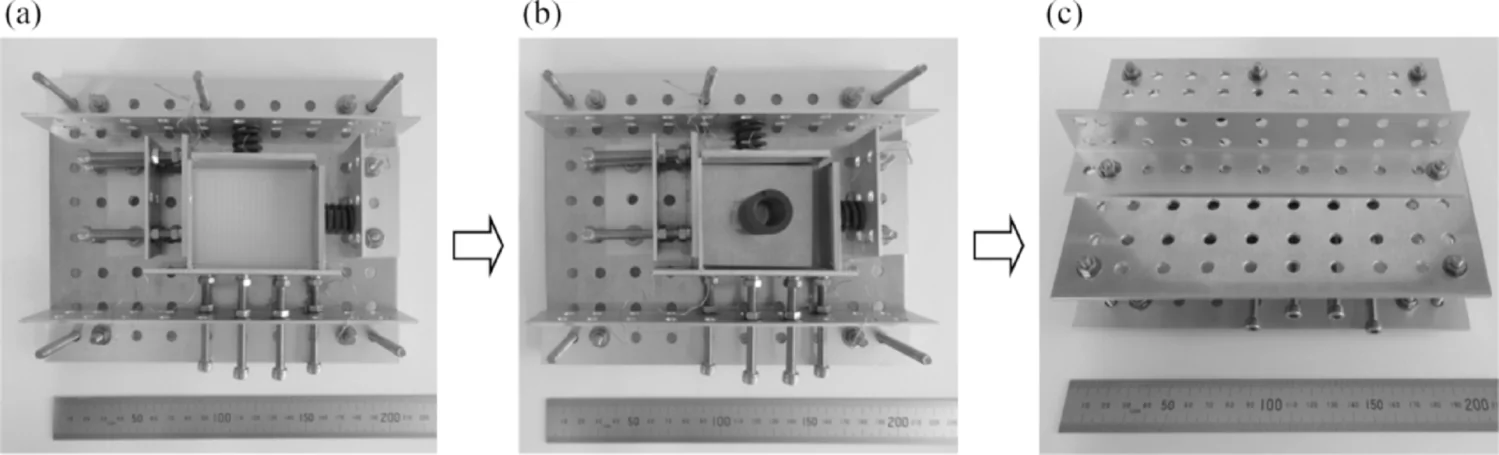
Schematic of placing fluidic chips in compression tool. **a** PP fluidic chip was placed in the compression tool. Stainless steel wires were connected to the parts around the fluidic chip in case the parts pop out. **b** An aluminum plate was placed on top of the fluidic chip, and a spring was fixed to the aluminum plate with double-sided tape. **c** An aluminum plate was placed on top of it, and the aluminum plate was fixed with bolts and nuts

Photographs of the procedure for placing the fluidic chips in the compression tool are shown in Fig. 1. When placing the fluidic chips, stainless steel wires were attached to the surrounding parts around the fluidic chip in case any parts popped out. After the PP fluidic chip was placed in the compression tool, it was heated in an electric furnace (FO310, Yamato Scientific Co., Ltd.) at 135 °C for approximately 1 h. After compression, the cross section of the channel was exposed by cutting the corner of the fluidic chip. PTFE tubing (outer diameter: 1/16, inner diameter: 1.0 mm) was thinned by pulling. A thinned section of PTFE tubing was then inserted into the channel and fixed using a two-Component epoxy adhesives (CEMEDINE Co., Ltd.).

### Solvent-based post-processing of the channel inside the polypropylene fluidic chip

In this study, solvent-based post-processing was performed on the channels inside the PP fluidic chip after the compression process. A schematic of the solvent-based post-processing of the channel inside the PP fluidic chip was shown in Fig. S3 (Supporting Information).　A PP fluidic chip was fixed to a 3 mm thick aluminum plate using double-sided tape (Nichiban Co., Ltd). This aluminum plate acted as a weight to prevent the chip from floating when placed in water. The PP fluidic chip was placed in triple-layered polyethylene bags, and then immersed in boiling water in a water bath (the thickness of one layer of polyethylene bag was approximately 20 μm). The fluidic chip was heated for approximately 20 min. After this heating steps, while the chip remained hot (i.e., at the temperature of the boiling-water bath), heptane was injected into the channel using a syringe. The heptane that passed through the flow path became a slightly white suspension. Using two air pumps, air was continuously introduced into the channel for approximately 30 min while the fluidic chip was maintained at an elevated temperature (i.e., at the temperature of the boiling-water bath). This solvent-based post-processing was carried out in a draft chamber.

After solvent-based post-processing, the PTFE tubing connected to the chip was replaced with components made of different materials. Although epoxy adhesives and PTFE tubing exhibit some resistance to hot water, and were therefore used during post-processing, epoxy adhesives show poor adhesion to polypropylene and PTFE, which can result in leakage at the bonded interfaces.

The epoxy-bonded section and PTFE tubing were removed, and a polyethylene tubes (Hibiki, Size No. 4, outer diameter approximately 1.33 mm) were attached. While a small amount of air was supplied to the chip using an air pump, the polyethylene tubes were partially fixed using Aron Alpha Plastic Adhesive (TOAGOSEI Co., Ltd., Product No. 32114). Subsequently, without air supply, the polyethylene tubes were firmly fixed using the same adhesive.

### Solvent-based post-processing outside the polypropylene fluidic chip

A schematic diagram and photograph of the tool used for solvent-based post-processing outside the PP fluidic chip are shown in S4 (Supporting Information). An aluminum mesh was placed inside a beaker. A watch glass was then placed convex side up on the mesh, and another watch glass was placed convex side down on top of the beaker. Heptane was poured into the beaker, which was then placed on a mantle heater. Prior to heating, aluminum foil was placed over the top surface of the heater, and the gap between the mantle heater and the beaker was filled with aluminum tart stones. The beaker was heated until evaporated heptane filled its interior. The watch glasses were then removed, and the fluidic chip was placed inside the aluminum mesh. A watch glass was again placed convex side up on the mesh, and another was placed convex side down on the beaker.

After post-processing the surface of the fluidic chip with heptane vapor, the beaker was removed from the mantle heater. As the beaker was hot, gloves were worn during handling. The two watch glasses were removed, and the beaker was allowed to cool at room temperature. After sufficient cooling, the fluidic chip was taken out from the mesh. This solvent-based post-processing was carried out in a draft chamber.

### Flow-injection measurements system

The Liquid flow rate was controlled using a syringe pump (KD Scientific, USA) and HPLC pump (Intelligent pump UI-22–110 S, FLOM Corporation). An absorption detector (SEC 2000, BAS Co., Ltd.) was used. Graphs were generated using Excel. The flow cell used for these measurements is described in the Supporting Information of a previous report [25].

### Measurement of Allura red AC

The carrier solution was delivered at a flow rate of 0.5 mL/min by HPLC pump. A 50% ethanol–water solution was used as the carrier. This 50% (v/v) ethanol–water solution was prepared by placing 100 mL of ethanol into a 200 mL volumetric flask and diluting to volume with water. The sample solution was prepared by dissolving and diluting Allura Red AC in a 50% (v/v) ethanol–water solution. The injection volume was 0.1 mL. The detection wavelength for absorbance measurements was 510 nm. In this experiment, a chip post-processed with 20 mL of heptane in its internal channel was used.

### Measurement of methyl orange

The carrier solution was delivered at a flow rate of 0.3 mL/min by syringe pump. An aqueous 0.1 M hydrochloric acid solution was used as the carrier. The sample solution (1 × 10^− 4^ M methyl orange) was prepared by diluting a 3.06 × 10^− 4^ M methyl orange aqueous solution with approximately 1 × 10^− 3^ M aqueous sodium hydroxide. The injection volume was 0.1 mL. The detection wavelength for absorbance measurements was 510 nm. In this experiment, a chip post-processed with 20 mL of heptane in its internal channel was used.

## Results and discussion

### 3D printing of polypropylene chip

A schematic of the fabrication of fluidic chips by 3D printing is shown in Fig. S5 (Supporting Information). Generally, 3D printing of PP is more challenging than that of other materials because of its poor adhesion to the print stage. In this study, PP sheets were attached to the print stage prior to printing, as molten PP filament adheres well to PP surfaces. However, the PP sheet often adhered too strongly and could not be removed. In such cases, the PP sheet was cut along the outline of the fluidic chip.

**Fig. 2:**
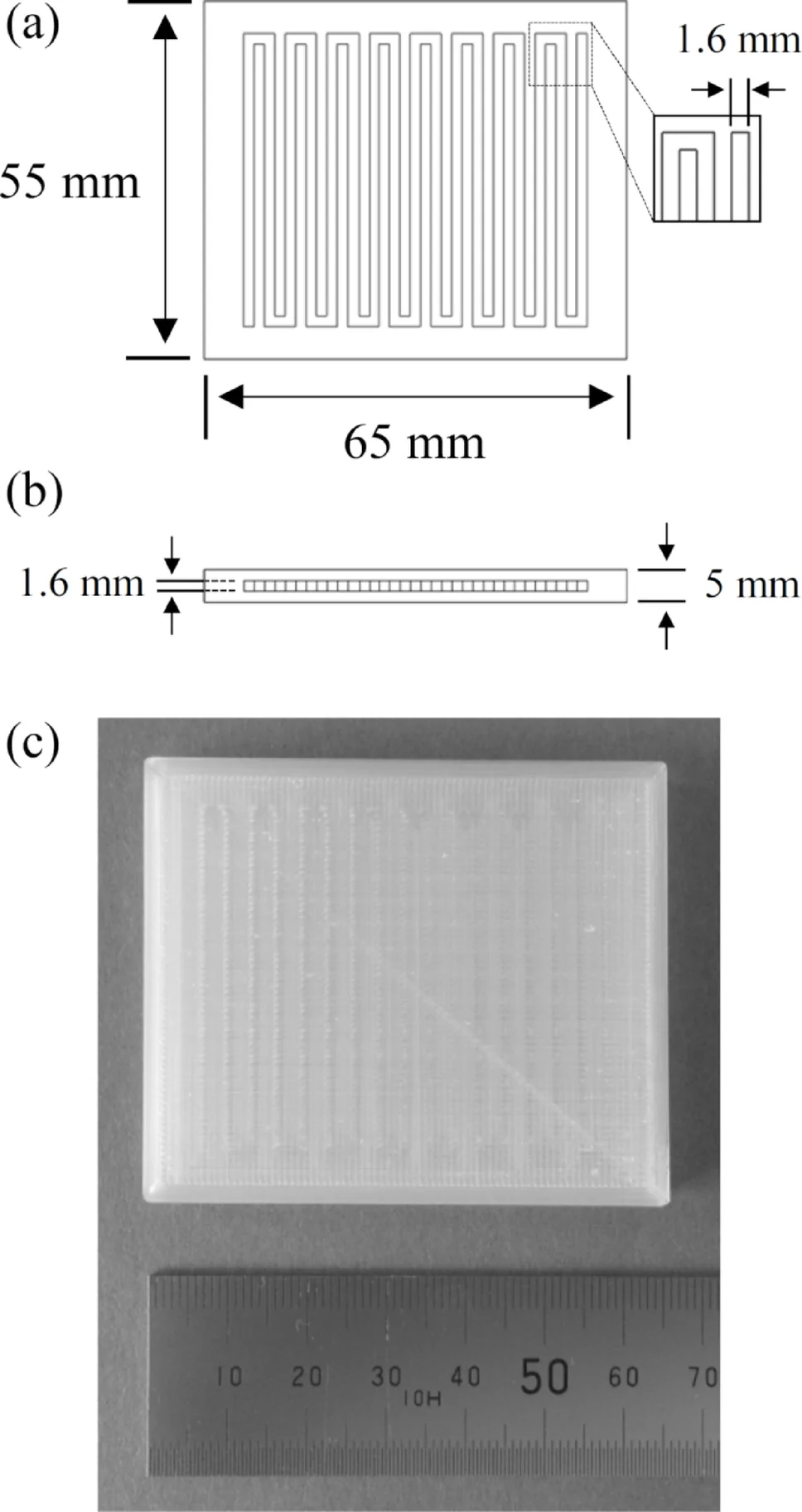
**a** Top perspective view of 3D model of fluidic chip (Brightness and contrast are adjusted), **b** Cross sectional view of 3D model of 3D chip (Brightness and contrast are adjusted), **c** Picture of fluidic chip made by 3D printer

The completed 3D model and the fabrication process are shown in Fig. 2 and Fig. S6 (Supporting Information), respectively. When PP is used for 3D printing, stringing is likely to occur.　To reduce the effect of stringing, several adjustments were made to the 3D model design and printing process.　(1) The fluidic chip was designed without an inlet or outlet for the channel to simplify the geometry and, consequently, the nozzle movement during printing. (2) The design was optimized to minimize nozzle movement over the channel region during non-printing travel.　This is because molten PP tends to drip into the channel when the nozzle moves over this region without extrusion.　As a specific strategy, the wall thickness between channels was set to an even multiple of the nozzle diameter. Although based on empirical observation, this approach appears to guide the slicer software to calculate nozzle paths that avoid crossing the channel region. Examples of slicer calculated paths for different channel wall thicknesses are shown in Fig. S7 (Supporting Information).　The nozzle diameter used in this study was 0.4 mm.　When the channel wall thickness was an odd multiple (1.2 mm) of the nozzle diameter, the slicer software generated paths in which the nozzle moved over the channel region near the edge during non-printing travel. In contrast, when the wall thickness was an even multiple (0.8–1.6 mm) of the nozzle diameter, the calculated paths avoid movement over the channel. In this study, fluidic chips without an upper structure were fabricated specifically for this experiment. The printing results for wall thicknesses of 0.8, 1.2, and 1.6 mm are shown in Fig. S8 (Supporting Information). When the wall thickness was an odd multiple (1.2 mm) of the nozzle diameter, stringing of polypropylene within the channel was observed. In contrast, when the wall thickness was an even multiple (0.8 and 1.6 mm), stringing within the channel was observed only to a negligible extent. The toolpaths in this study were generated using MatterSlice as the slicer software.　(Path calculations using other slicers were not examined.)

Generally, in FFF-type 3D printers, the initial build height (distance between the print bed and the nozzle) is adjusted prior to printing. In this study, the initial build height was adjusted manually, and its setting affected the printing results. A comparison of channel inner walls printed under different initial build height settings is shown in Fig. S9 (Supporting Information). When the initial build height was small (adjusted to approximately the thickness of one sheet of paper between the nozzle and the print bed), the print quality was poor. In contrast, when the initial build height was large (adjusted to approximately the thickness of three sheets of paper), the print quality was generally good. However, in some cases, the initial build height was too large, resulting in insufficient adhesion of PP to the print bed. In this study, the initial build height was adjusted to a value corresponding approximately to the thickness of one to three sheets of paper. Because this adjustment range is narrow, manual adjustment makes it difficult to reproduce the same initial build height consistently, resulting in variability in print quality.

In this study, a straight‑edged channel geometry was employed. This geometry was selected because its simple, linear shape allows the printed surface to form more uniformly, which is advantageous for direct SEM observation of the surface before and after the smoothing process. For reference, the printing results obtained when the channel geometry was changed to a circular shape are provided in Fig. S10 (supporting information). The circular channels tended to exhibit slightly more irregular printing outcomes compared with the straight‑edged ones.

### Compression of fluidic chip

This study includes a leakage-reduction method in which gaps between layers are filled with PP through solvent-based post-processing. However, during this process, a sufficient amount of solvent must reach the outlet of the flow path. If the solvent leaks out before reaching the outlet, solvent-based post-processing of the entire channel cannot be achieved. In this study, a compression process was applied to minimize solvent leakage from the channel during post-processing. However, if 3D printing alone can produce a chip that allows solvent to pass through the entire channel, this compression process may not be required necessary.

In the compression process used in this study, the polymer first softened by heating and then lightly bonded. This concept has been reported in microfluidic studies for cyclic olefin copolymers [18]. PP exhibits softening behavior upon heating; therefore, compressing PP in its softened state is expected to enhance interlayer adhesion. In general, objects fabricated by 3D printing consist of layers oriented in multiple directions. Fig. S11 (Supporting Information) shows the slicer-software calculated internal structure of the channel in the fluidic chip (horizontal cross-sectional view). Because the channels are formed constructed by stacking layers oriented in various directions, it is desirable to apply compression along the X, Y, and Z axes. The compression tool used in this study is capable of applying compression along all three axes.

The compression tool is made of metal to withstand the high temperature of the furnace. The softening point of PP is generally around 110 °C, and its melting point is 170–180 °C. In this study, the fluidic chip was heated at 135 °C. At this temperature, PP softens but does not melt; therefore, the layers are not fully bonded by melting. However, compressing softened PP is expected to enhance interlayer adhesion. The force applied to compress the fluidic chip using the compression tool in this study was estimated to be in the range of 5–20 kg.

To investigate the effectiveness of the compression tool, an experiment was conducted in which eight test pieces were compressed. Fig. S12 (Supporting Information) shows the results of this experiment.　After compression, the eight test pieces were lightly adhered; however, they separated when a certain amount of force was applied. Therefore, the adhesive strength is considered weak.

The compression tool enhances interlayer adhesion to some extent, which is expected to help reduce leakage. However, even with the compression tool, the gaps are not completely closed. To further reduce leakage, solvent-based post-processing was applied to the fluidic chip. The results are described in the next section.

### Solvent-based post-processing of the channel inside the polypropylene fluidic chip

In this process, the inner walls of the PP channel were swollen or partially dissolved by solvent-based post-processing. As a result, the gaps between layers were filled with PP. Heptane was used as the solvent. An advantage of using heptane is that boiling water can be serve as the heating source for solvent evaporation, as the boiling point of heptane is approximately 98 °C.

**Fig. 3:**
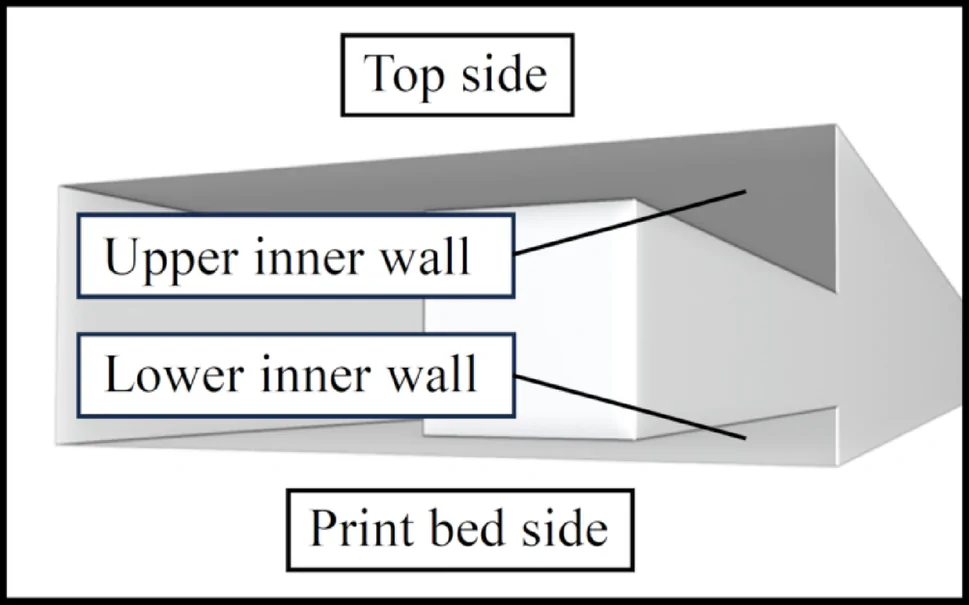
Illustration of the positions of the upper and lower inner walls in the channel

**Fig. 4:**
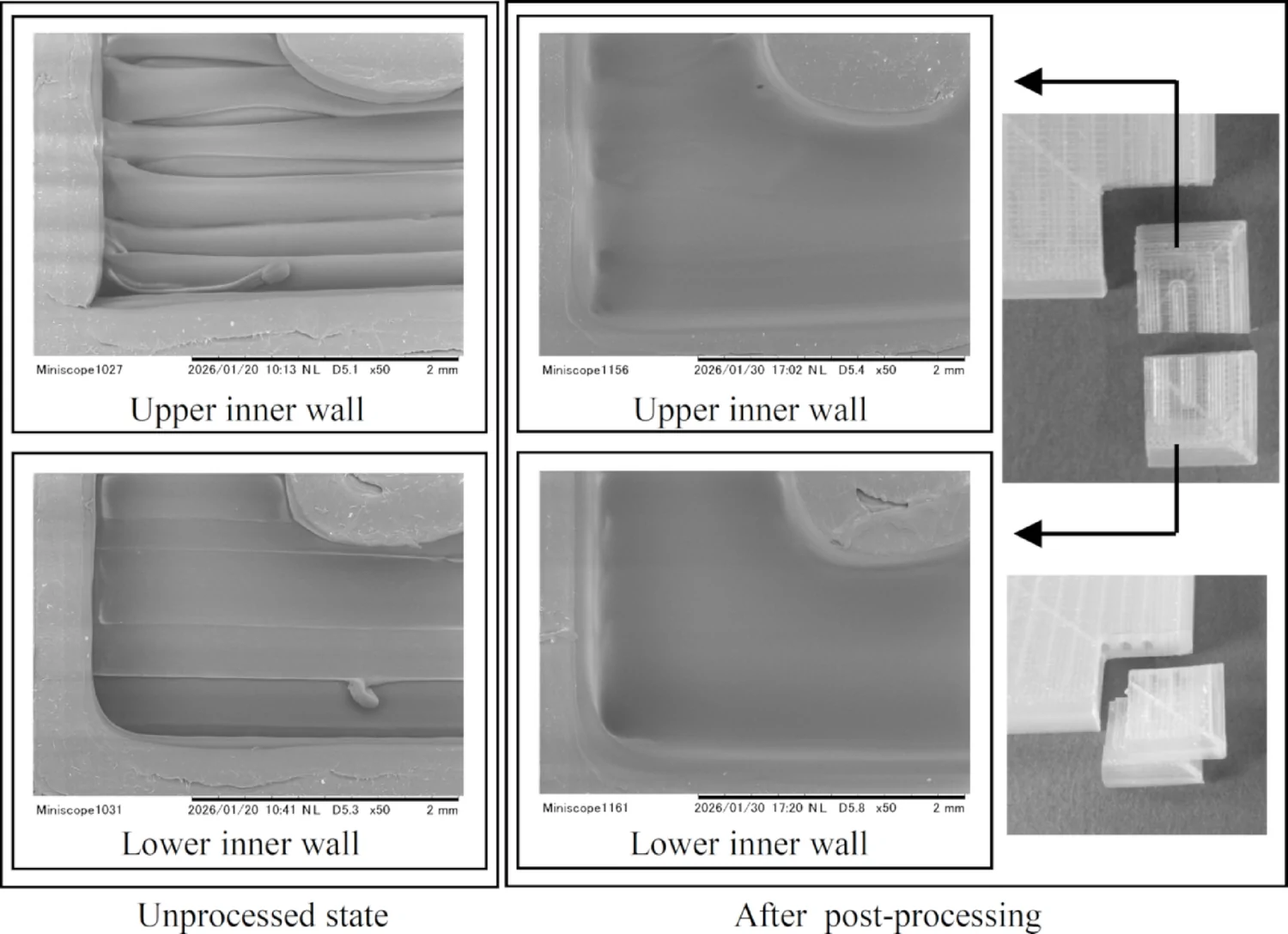
Scanning electron micrographs (50× magnification) of the inner wall of a PP fluidic chip in the unprocessed state and after post-processing with approximately 20 mL of heptane

Figure 3 shows the positions of the upper and lower inner walls in the channel, and Fig. 4 shows scanning electron micrographs of the inner walls of the PP fluidic chip in the unprocessed state and after post-processing with approximately 20 mL of heptane. The reason for using 20 mL of heptane is to ensure that the solvent comes into contact with the inner walls of the channel. After post-processing, the gaps between layers were smaller than those in the unprocessed state.

In Fig. 4, the gap in the upper inner wall is larger than that in the lower inner wall. The reason for this is considered as follows. An illustration explaining this is shown in Fig. S13 (Supporting Information). In layered regions, the extruded filament is pressed between the nozzle and the underlying layer, causing lateral spreading and reducing the gap between adjacent layers. In contrast, in overhang regions, no supporting surface exists beneath the extruded filament, and thus no compression occurs. Consequently, gaps between adjacent layers are larger.

However, when solvent-based post-processing was performed with approximately 20 mL of heptane, deposits were sometimes observed inside the channel. This phenomenon is presumed to result from excess solvent remaining in the channel. When the solvent containing dissolved PP evaporates, PP deposits are likely to form on the inner surfaces of the channel.

### Leakage tests for PP fluidic chips under different post‑processing conditions

Three chips were fabricated under each condition—compression‑processed only, solvent‑processed only, and no compression or solvent processing—resulting in a total of nine chips. Each chip was first filled with water and ethanol and left to stand for 5 min to check for leakage. Subsequently, water and ethanol were flowed through the channels at various flow rates for 1 min, and the presence or absence of leakage was examined. A summary of the experimental results is provided in Fig. S14 (Supporting Information). No leakage was observed for any of the chips. These findings indicate that polypropylene chips fabricated under the 3D printing conditions used in this study possess a certain degree of leakage resistance.

To show what happens when leakage occurs, a chip intentionally fabricated to leak was produced by setting the extrusion multiplier to 0.9, thereby making gaps between layers during 3D printing. A photograph of the leakage from this chip is provided in Fig. S15 (Supporting Information). In addition, in some cases, leakage was observed from the adhesive used to connect the tubing.

### Solvent-based post-processing of outside surface of the polypropylene fluidic chip

**Fig. 5:**
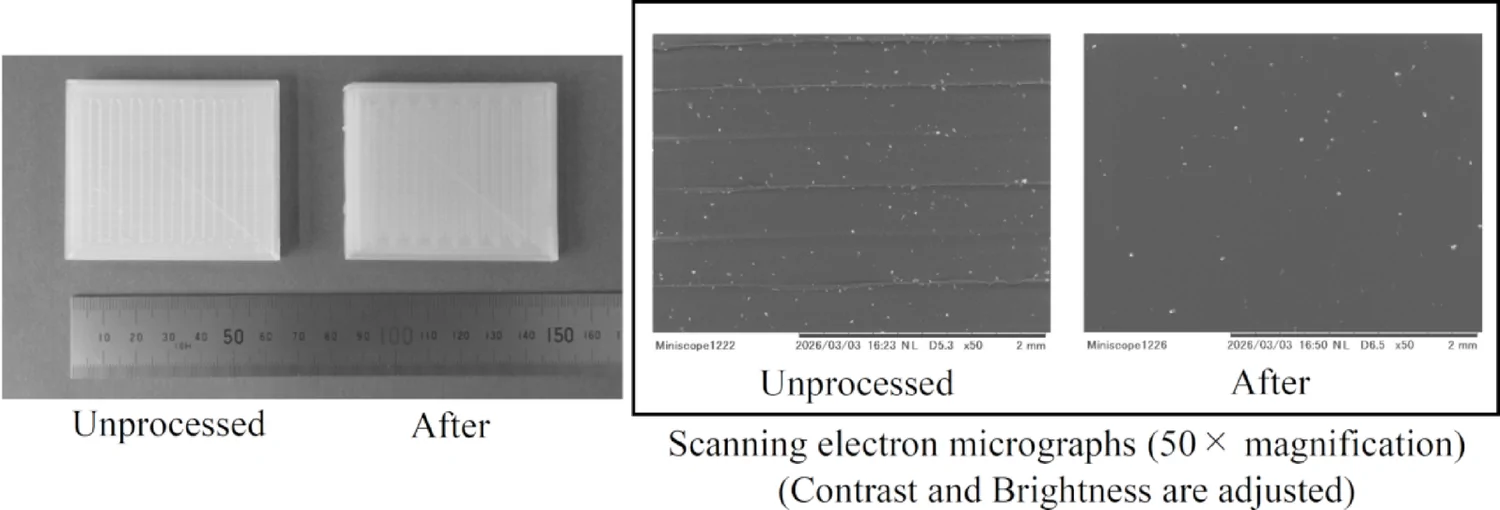
Photographs of the outer surface of a PP fluidic chip in the unprocessed state and after post-processing (fluidic chips without the compression process were used in this experiment)

Solvent-based post-processing of the outer surface of the chip is also possible. In this study, post-processing was performed only on the outer surface of the fluidic chip using heptane. Figure 5 shows photographs and scanning electron micrographs of the outer surface of a PP fluidic chip in the unprocessed state and after solvent-based post-processing (in this experiment, fluidic chips without the compression process were used).　After post-processing, the chip surface became smoother, and the gaps between layers are considered to have been filled.

Post-processing of the outer surface is not directly related to preventing leakage in a polypropylene fluidic chip. However, this method can be applied to other 3D-printed objects. For example, when 3D-printed containers such as flasks or bottles are used to hold liquids, post-processing of the outer surface can help improve their leak resistance.

### Application of a polypropylene fluidic chip for flow-injection measurements using a 50% (v/v) ethanol–water carrier solution

**Fig. 6:**
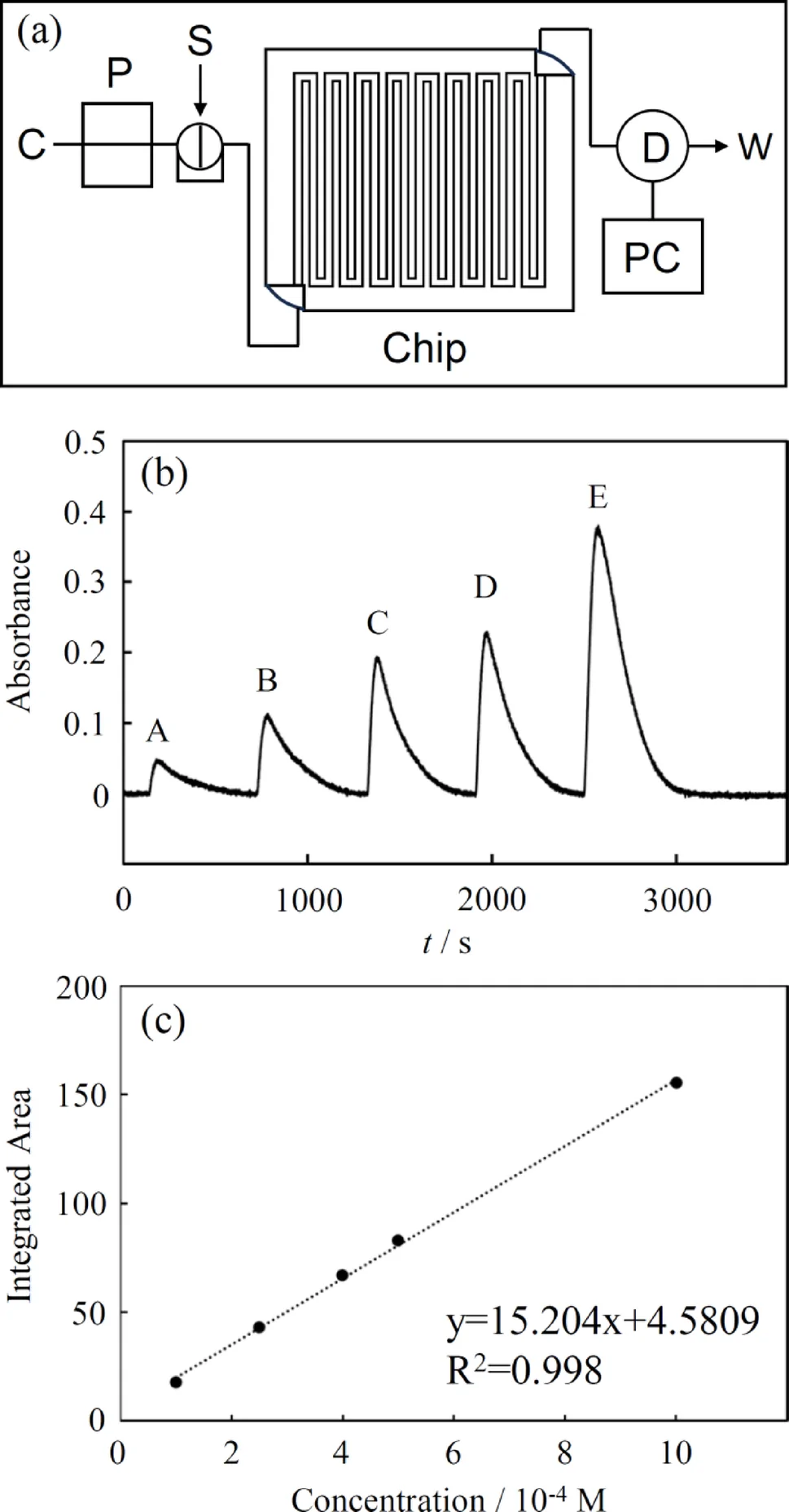
**a** A schematic illustration of the flow injection system for measurement of Allura Red AC: C, carrier solution (50% (v/v) ethanol–water solution); P, HPLC pump; S, sample injector; D, detector; PC, personal computer; W, waste. **b** Flow signals for the calibration graph of phosphate: A, 1×10^-4^ M; B, 2.5×10^-4^ M; C, 4×10^-4^ M; D, 5×10^-4^ M, E, 10.02×10^-4^ M after post‑processing with approximately 20 mL of heptane. **c** Calibration curve with linear fitting

To evaluate the usability of the post‑processed chip, it was used to measure the flow profile of Allura Red AC in a 50% (v/v) ethanol–water solution. A schematic illustration of the flow‑injection system is shown in Fig. 6a, and photographs of the flow‑injection analysis setup and the post‑processed PP chip are shown in Fig. S16a (Supporting Information). Photographs of the flow‑injection analysis setup without PP chip are shown in Fig. S16b (Supporting Information). A schematic illustration of the flow‑injection system used to measure the flow profile of Allura Red AC without the fluidic chip, along with the resulting flow profile, is shown in Fig. S17 (Supporting Information). In this experiment, a fluidic chip post‑processed with approximately 20 mL of heptane was used. The flow signals and calibration curve are shown in Fig. 6b and c, respectively, and the absorption spectrum of Allura Red AC solution is shown in Fig. S18a (Supporting Information). The calibration curve exhibited good linearity. To assess the stability of the measurements, repeated‑injection experiments were performed. The results are shown in Fig. S19a (Supporting Information), and the coefficient of variation (CV) calculated from these measurements is shown in Fig. S19b (Supporting Information) (the concentrations were determined using the calibration curve in Fig. 6c). The results confirmed that stable continuous measurements could be achieved. During the measurements, no leakage from the chip was observed. These results shows that a PP chip can be used for flow‑injection analysis.

### Application of a polypropylene fluidic chip for flow-injection measurements using an aqueous HCl carrier solution

PP has the advantage of being resistant to both acids and bases. In this study, post‑processed chip was also used to measure the flow profile of methyl orange using an aqueous HCl carrier solution. A schematic illustration of the flow-injection system is shown in Fig. S20a (Supporting Information). Fig. S21a (Supporting Information) shows photographs of the flow-injection analysis system and the post-processed PP chip. In this experiment, a fluidic chip post-processed with approximately 20 mL of heptane was used. The absorption spectrum of methyl orange solution is shown in Fig. S18b (Supporting Information). The resulting flow profile is shown in Fig. S20b (Supporting Information). Peak profiles were obtained, and no leakage from the chip was observed during the measurements.

A schematic illustration of the flow‑injection system used to measure the flow profile of methyl orange without the fluidic chip, along with the resulting flow profile, is shown in Fig. S22 (Supporting Information). Photographs of the flow‑injection analysis setup without PP chip are shown in Fig. S21b (Supporting Information). In this measurement setup, the peak time of the flow profile becomes shorter than that in Fig. S20b (Supporting Information) because the absence of the fluidic chip reduces the total flow‑path length.

## Conclusions

This study presented a new method for filling interlayer gaps on the inner surfaces of polypropylene fluidic channels fabricated using an FFF 3D printer by applying a solvent‑based post‑processing treatment. The post-processed chip was then used to measure peak profile in a flow-injection experiment. Peak profile was obtained, and no leakage from the chip was observed during the measurements. This method is expected to enable the filling of interlayer gaps on the inner surfaces of newly 3D‑printed polypropylene experimental tools with narrow and complex internal structures. 

## Supporting information

Parts of the compression tool are shown in Fig. S1. Summary of the 3D printing parameters used for fabricating the polypropylene fluidic chips are shown in Fig. S2. A schematic of solvent-based post-processing of the channel inside the PP fluidic chip is shown in Fig. S3. A schematic diagram and photograph of the tool used for solvent-based post-processing of the outer surface of the PP fluidic chip are shown in Fig. S4. Photographs of the fluidic chips fabricated using 3D printing are shown in Fig. S5. Schematic the fabrication of the 3D model of the fluidic chip is shown in Fig. S6. Examples of slicer software-calculated results for different channel wall thicknesses near the edge are shown in Fig. S7. Print results for different wall thicknesses are shown in Fig. S8. A comparison of channel inner walls printed under different initial build height settings is shown in Fig. S9. 3D models of the channel designed with a circular geometry and SEM images of the unprocessed channel are shown in Fig. S10. The result of a slicer software calculation of the internal channel structure of a fluidic chip is shown in Fig. S11. A schematic of the compression experiment is shown in Fig. S12. An explanation of the speculated gap difference is shown in Fig. S13. Polypropylene fluidic chips used for leakage testing, along with the leakage‑test results under static and flow conditions, are shown in Fig. S14. A photograph of a PP chip that was 3D‑printed with an extrusion multiplier of 0.9, showing leakage after water injection into the channel (no compression or solvent‑based post‑processing), is shown in Fig. S15. Photographs of the flow‑injection analysis system using an HPLC pump, with and without the post‑processed PP chip (a chip post‑processed with approximately 20 mL of heptane was used), are shown in Fig. S16. A schematic illustration of the flow‑injection system using an HPLC pump to measure the flow profile of Allura Red AC without the fluidic chip, together with the corresponding flow profiles of 5 × 10⁻⁴ M Allura Red AC, is shown in Fig. S17. Absorption spectra of 5 × 10⁻⁶ M Allura Red AC in 50% (v/v) ethanol–water solution and 1 × 10⁻⁵ M methyl orange in 0.1 M aqueous solution are shown in Fig. S18. Repeated injections of 5 × 10⁻⁴ M Allura Red AC and the calculation of the coefficient of variation (CV) are shown in Fig. S19. A schematic illustration of the flow‑injection system for measuring methyl orange and the corresponding flow profiles obtained using a chip post‑processed with approximately 20 mL of heptane are shown in Fig. S20. Photographs of the flow‑injection analysis system using a syringe pump, with and without the post‑processed PP chip (a chip post‑processed with approximately 20 mL of heptane was used), are shown in Fig. S21. A schematic illustration of the flow‑injection system using a syringe pump to measure the flow profile of methyl orange without the fluidic chip, together with the corresponding flow profiles of 1 × 10⁻⁴ M methyl orange in 1 × 10⁻³ M NaOH, is shown in Fig. S22.

## Supplementary Information

Below is the link to the electronic supplementary material.

Supplementary Material 1

## Data Availability

The authors declare that the data supporting the findings of this study are available within the paper and its Supplementary Information files. Should any raw data files be needed in another format they are available from the corresponding author upon reasonable request.
