# Supplementary material for: Solvent-based post-processing to fill layer gaps in FFF-printed polypropylene fluidic chips and their application to flow injection analysis
Source: Anal Sci. 2026 Aug 3;42(10):965–74. doi: 10.1007/s44211-026-00954-6 (PMC13588886; doi:10.1007/s44211-026-00954-6)
Supplement: Supplementary file 1 [file 44211_2026_954_MOESM1_ESM.pdf]

Supporting Information

Solvent-based post-processing to fill layer gaps in FFF-printed polypropylene fluidic chips and their application to flow injection analysis

Tomohisa YAMASHITA*†

Toyama Institute of Health, 17-1 Nakataikoyama, Imizu, Toyama 939-0363, Japan.

† To whom correspondence should be addressed.
E-mail: tomohisa.yamashita@pref.toyama.lg.jp

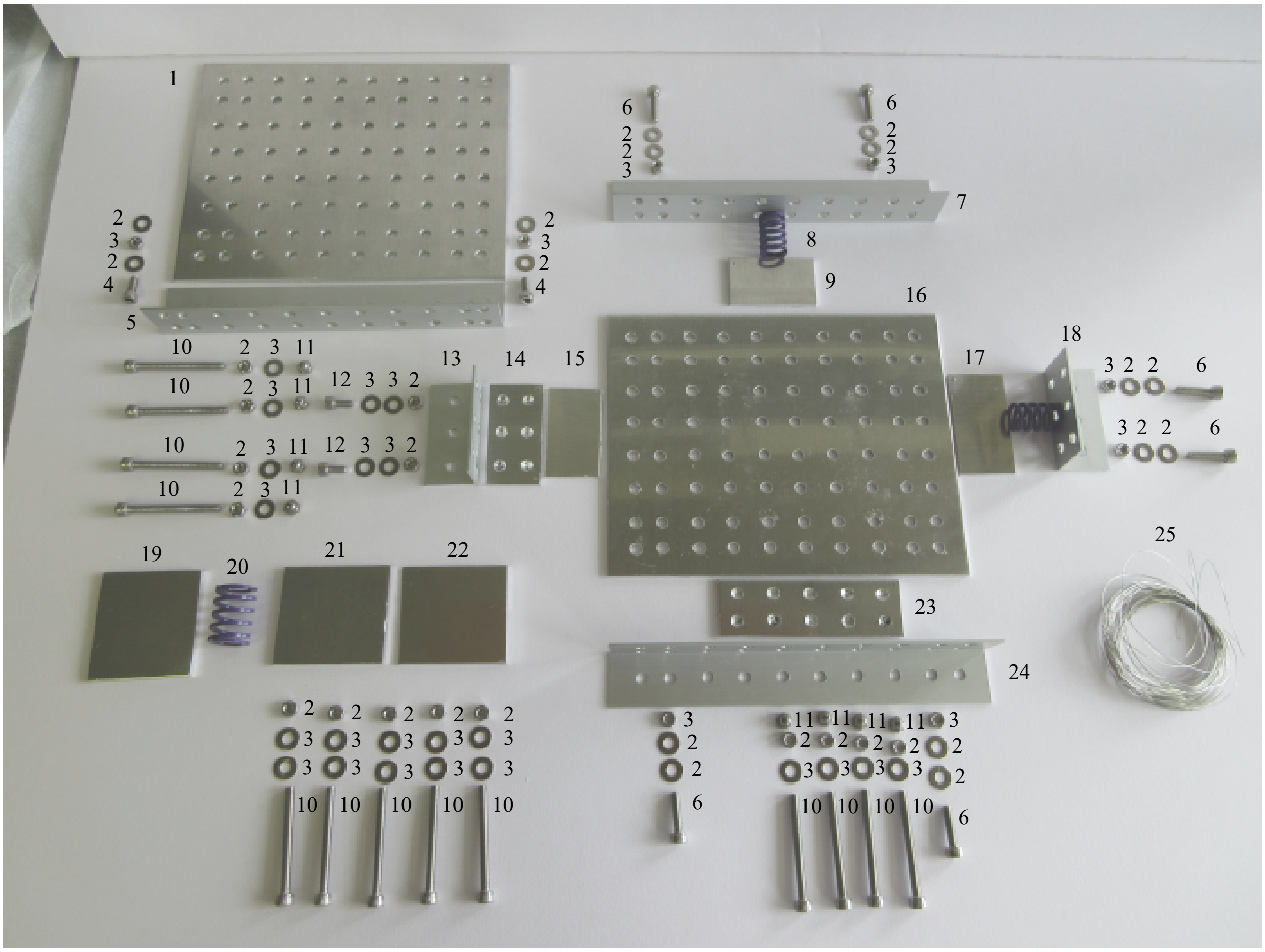

Fig. S1 Parts of the compression tool.
(Photograph after brightness and contrast adjustment)

1. Aluminum plate (3 mm thick)
2. Stainless steel washer
   (inner diameter M5, outer diameter 12 mm)
3. Stainless steel nut (M5)
4. Stainless steel bolt
   (M5, thread length 12 mm)
5. Aluminum angle (2 mm thick)
6. Stainless steel bolt
   (M5, thread length 20 mm)
7. Aluminum angle (2 mm thick)
8. Spring
   (Length 25 mm, diameter 17 mm)
9. Aluminum plate (3 mm thick)
10. Stainless steel bolt
    (M5, thread length 50 mm)
11. Stainless steel cap nut (M5)
12. Stainless steel bolt
    (M5, thread length 10 mm)
13. Aluminum angle (2 mm thick)
14. Aluminum plate (3 mm thick)
15. Aluminum plate (2 mm thick)
16. Aluminum plate (3 mm thick)
17. Aluminum plate (3 mm thick)
18. Aluminum angle (2 mm thick)
19. Aluminum plate (3 mm thick)
20. Spring
    (Length 30 mm, diameter 20 mm)
21. Aluminum plate (3 mm thick)
22. Aluminum plate (3 mm thick)
23. Aluminum plate (3 mm thick)
24. Aluminum angle (2 mm thick)
25. Stainless steel wire
    (Diameter 0.3 mm)

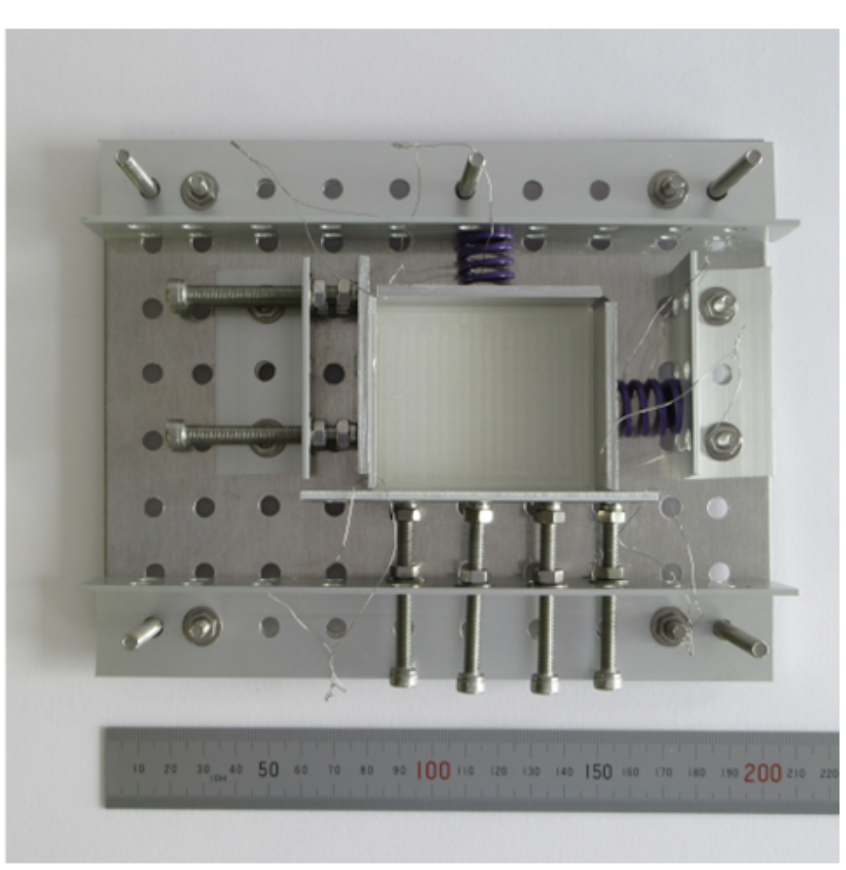

Photo of compression tool being assembled

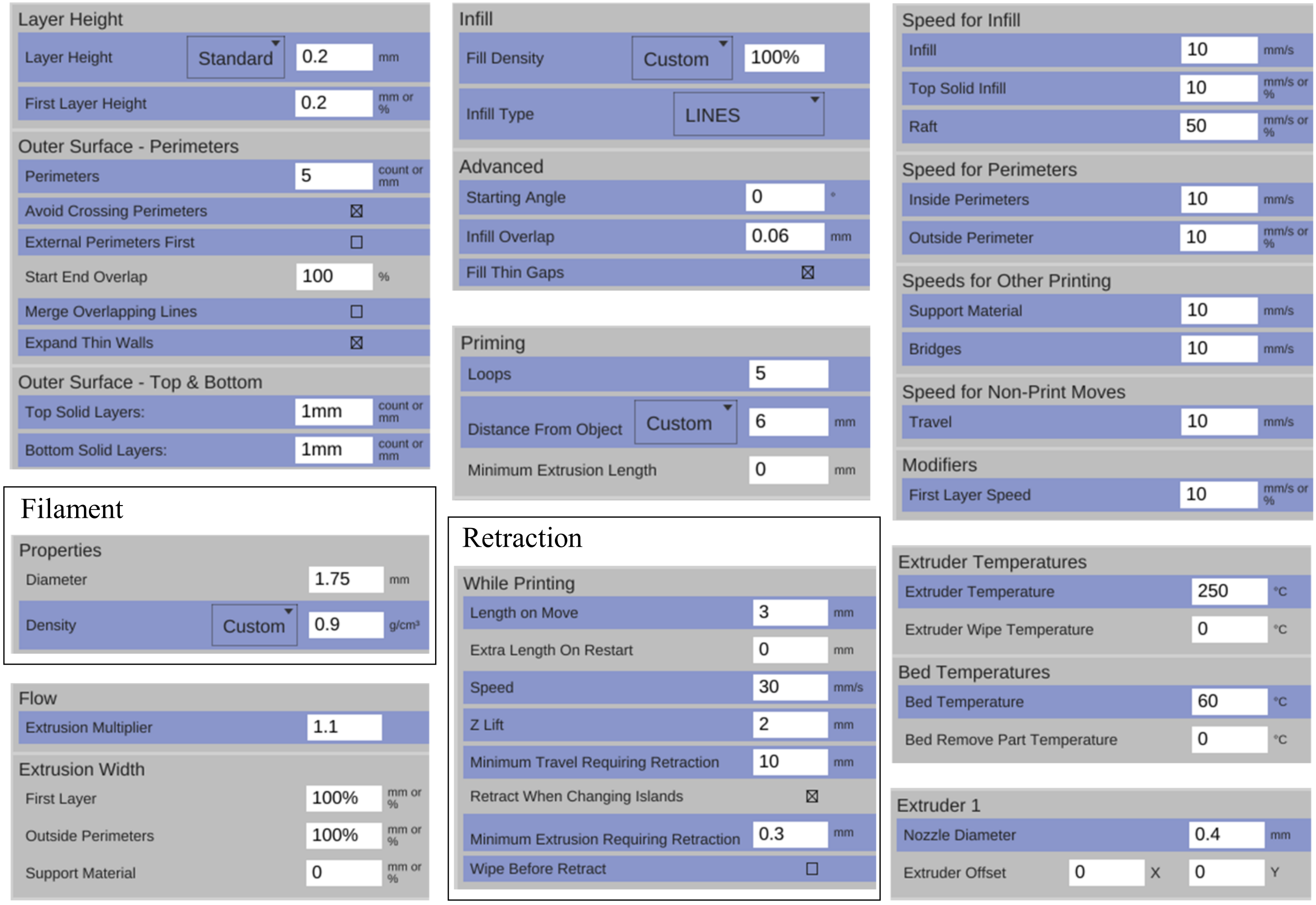

Fig. S2 Summary of the 3D printing parameters used for fabricating the polypropylene fluidic chips.

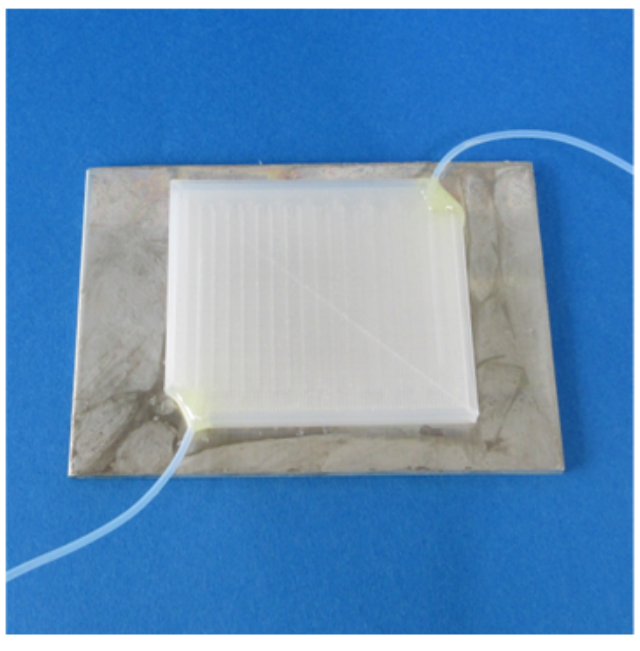

(a) A PP fluidic chip was fixed to a 3 mm thick aluminum plate using double-sided tape.

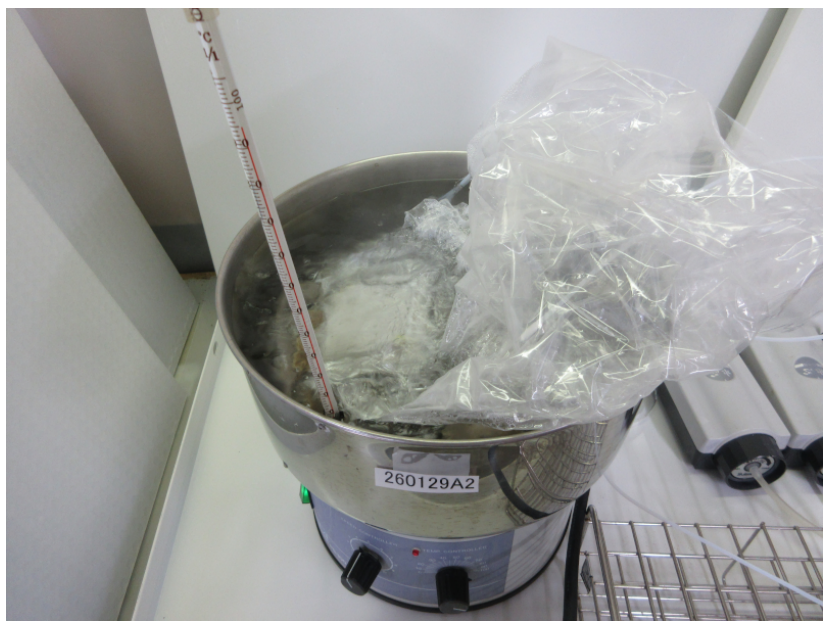

(b) A PP fluidic chip was placed in triple-layered polyethylene bags, and this polyethylene bag was placed in boiling water in a water bath.

(c) While heating, heptane was injected into the channel using a syringe. The heptane that had passed through the flow path became slightly white suspended liquid.

(d) Air was continuously introduced into the channel using an air pump while heating the chip.

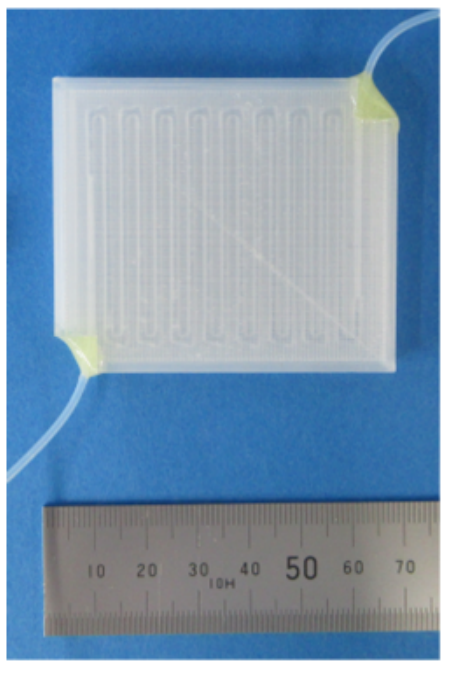

(e) PP fluidic chip after post-processing.

(f) The epoxy-bonded section and PTFE tubing were removed, and a polyethylene tube was attached and fixed using Aron Alpha Plastic Adhesive in multiple steps.

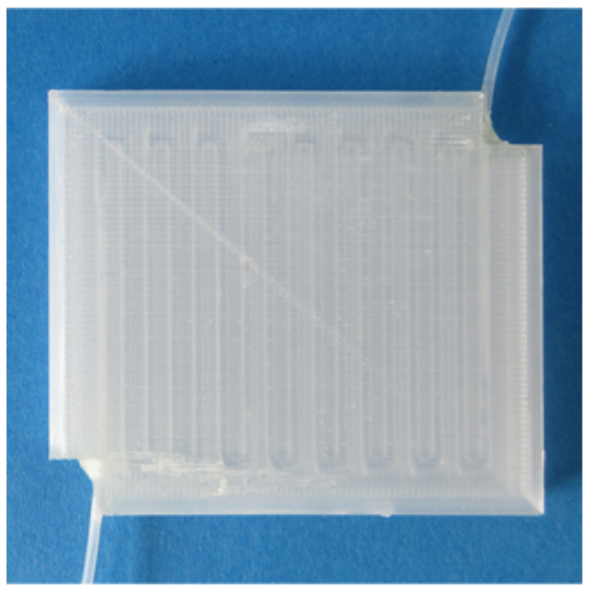

(g) PP fluidic chip after attachment of the polyethylene tube.

Fig. S3 Schematic of solvent-based post-processing of the channel inside the PP fluidic chip. (post-processing was carried out in a draft chamber.)

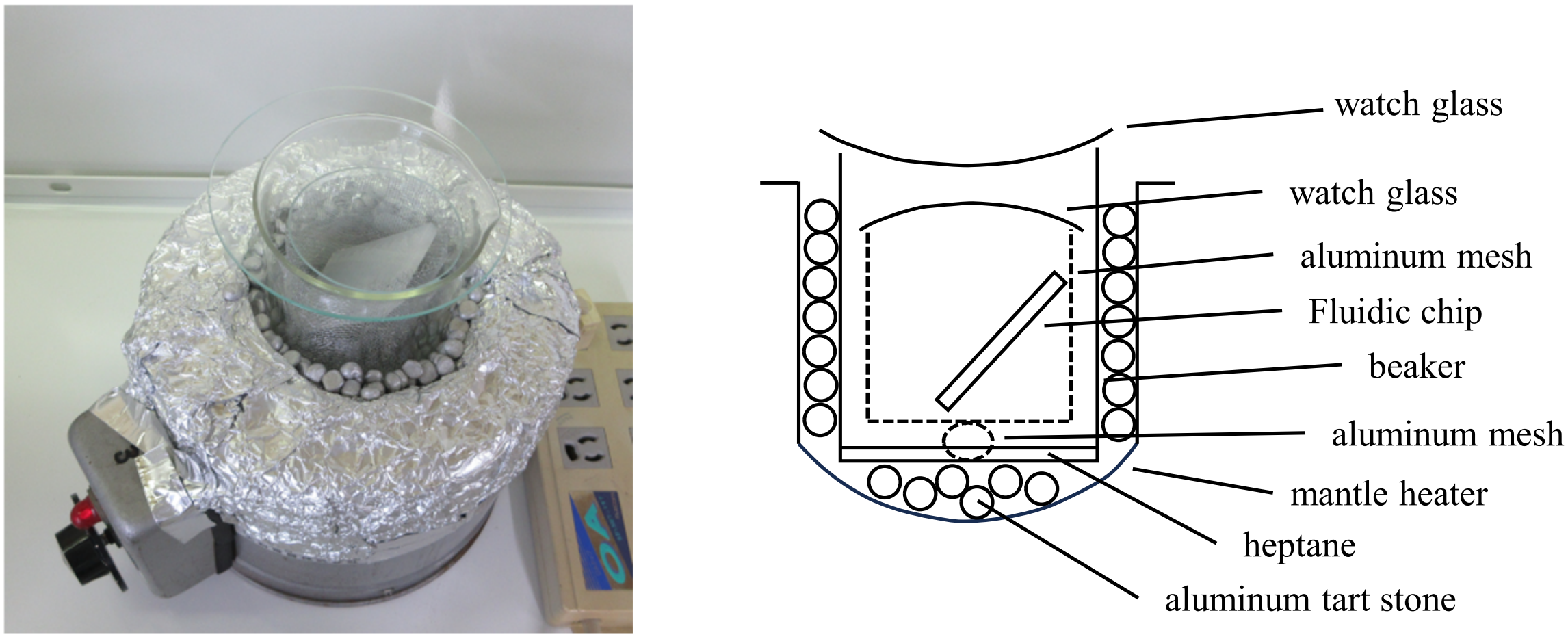

Fig. S4 Schematic diagram and photograph of the tool used for solvent-based post-processing of the outer surface of the PP fluidic chip (post-processing was carried out in a draft chamber.)

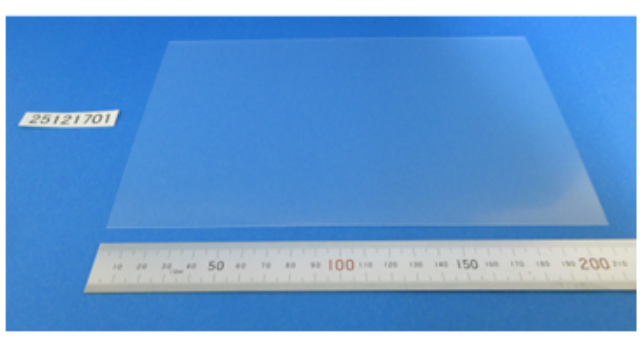

(a) PP sheet

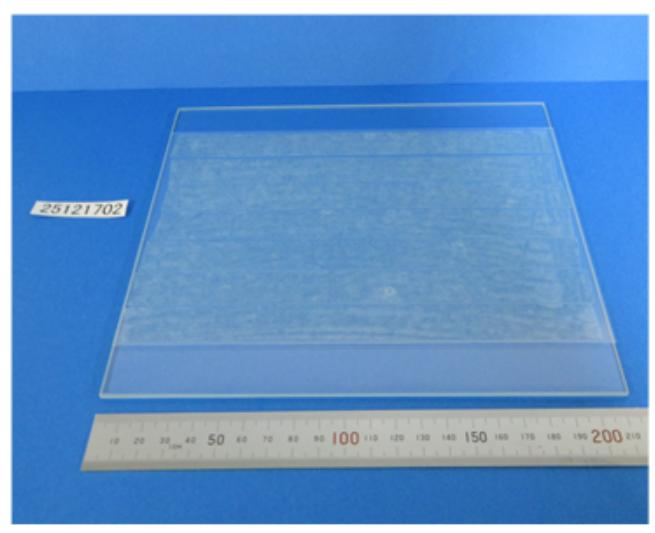

(b) Ultra Strong Double-Sided Adhesive Tape for PE / PP was applied on the print bed, and a PP sheet was placed on the tape.

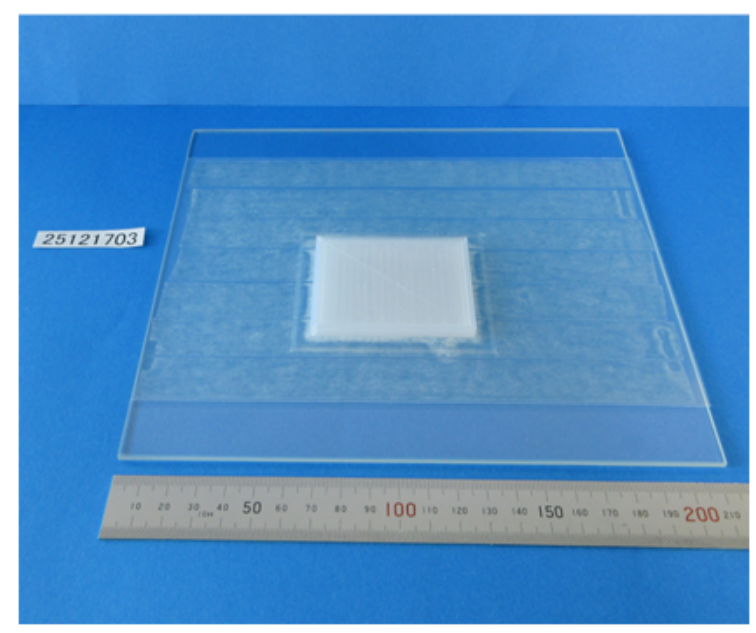

(c) Fluidic chip was fabricated on PP sheets.

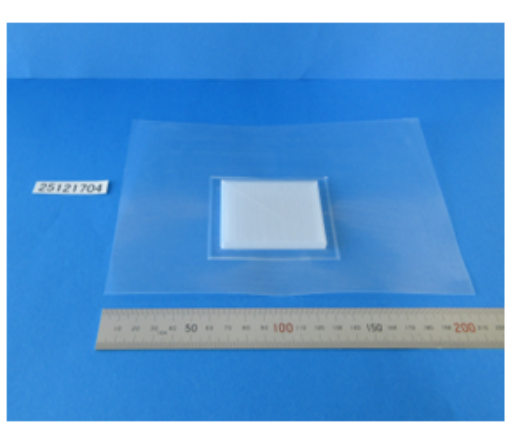

(d) PP sheet was taken off from the print bed.

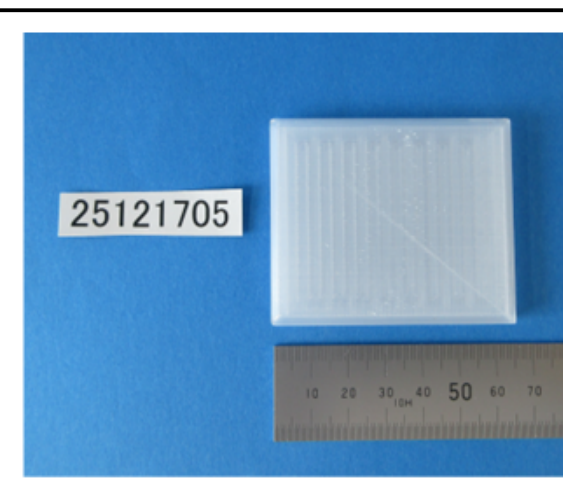

(e) The fluidic chips were taken from the PP sheet. (if the PP sheet adhered strongly and could not be taken off, it was cut along the outline of the fluidic chip).

Fig. S5 Photographs of the fluidic chips fabricated using 3D printing.

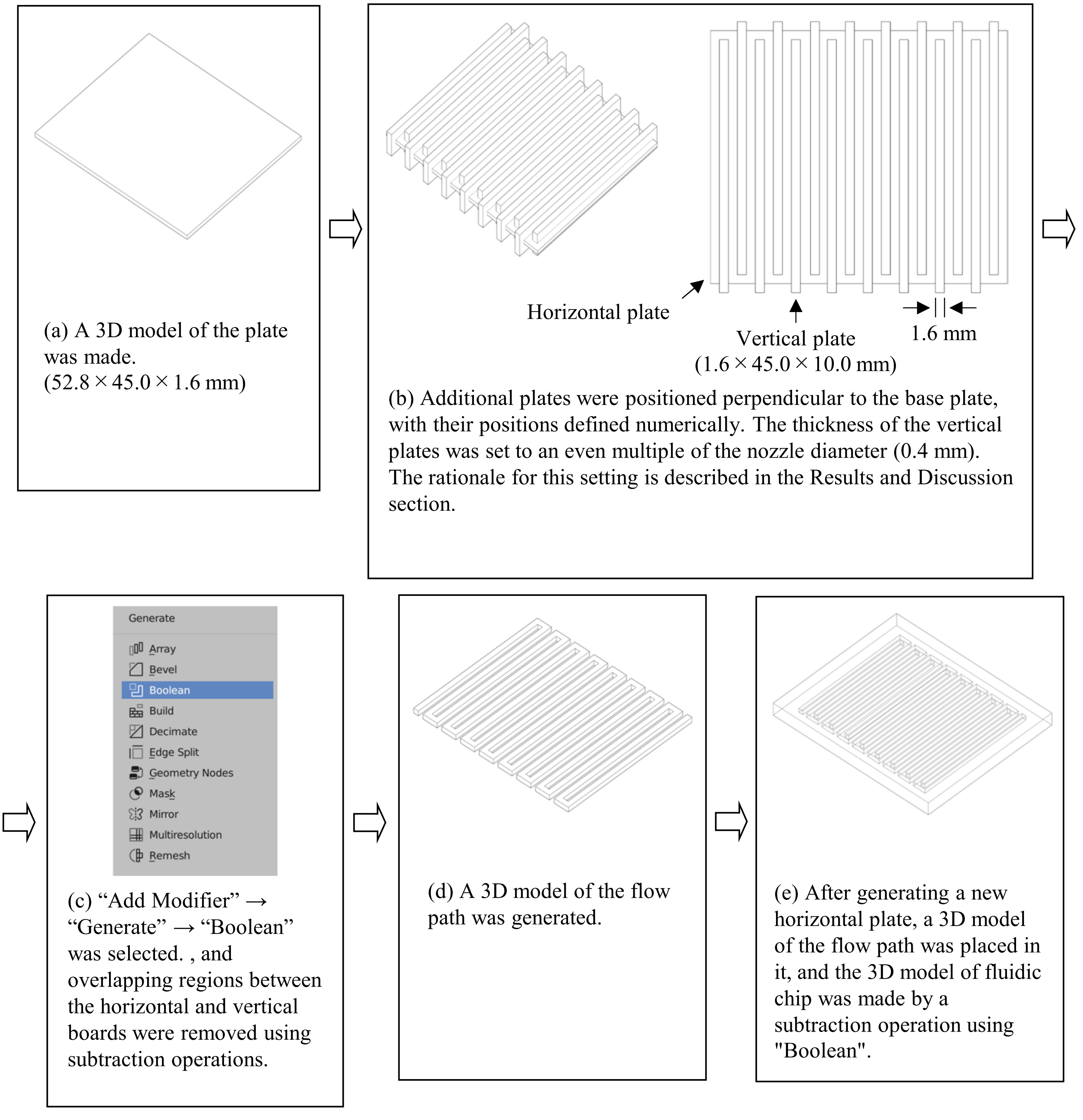

Fig. S6 Schematic the fabrication of the 3D model of the fluidic chip. Blender 2.93 (3D computer graphics software) was used.

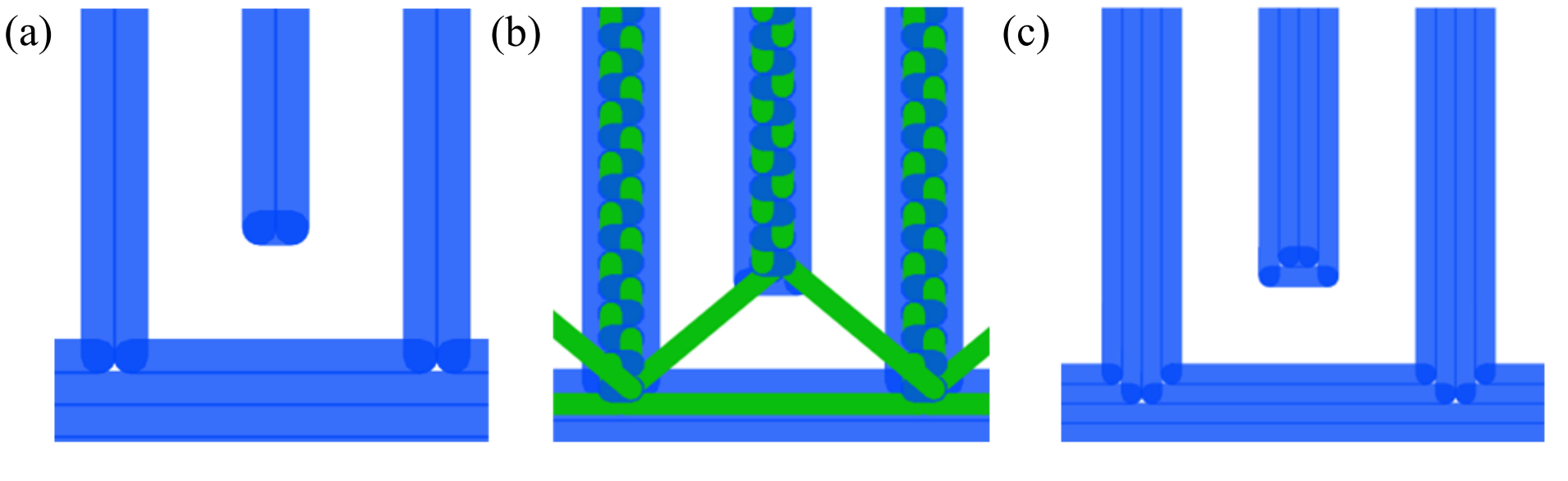

Fig. S7 Examples of slicer software–calculated paths for different channel wall thicknesses near the edge: (a) 0.8 mm, (b) 1.2 mm, and (c) 1.6 mm. The green line indicates non-printing nozzle movement. The nozzle diameter used in this study was 0.4 mm.

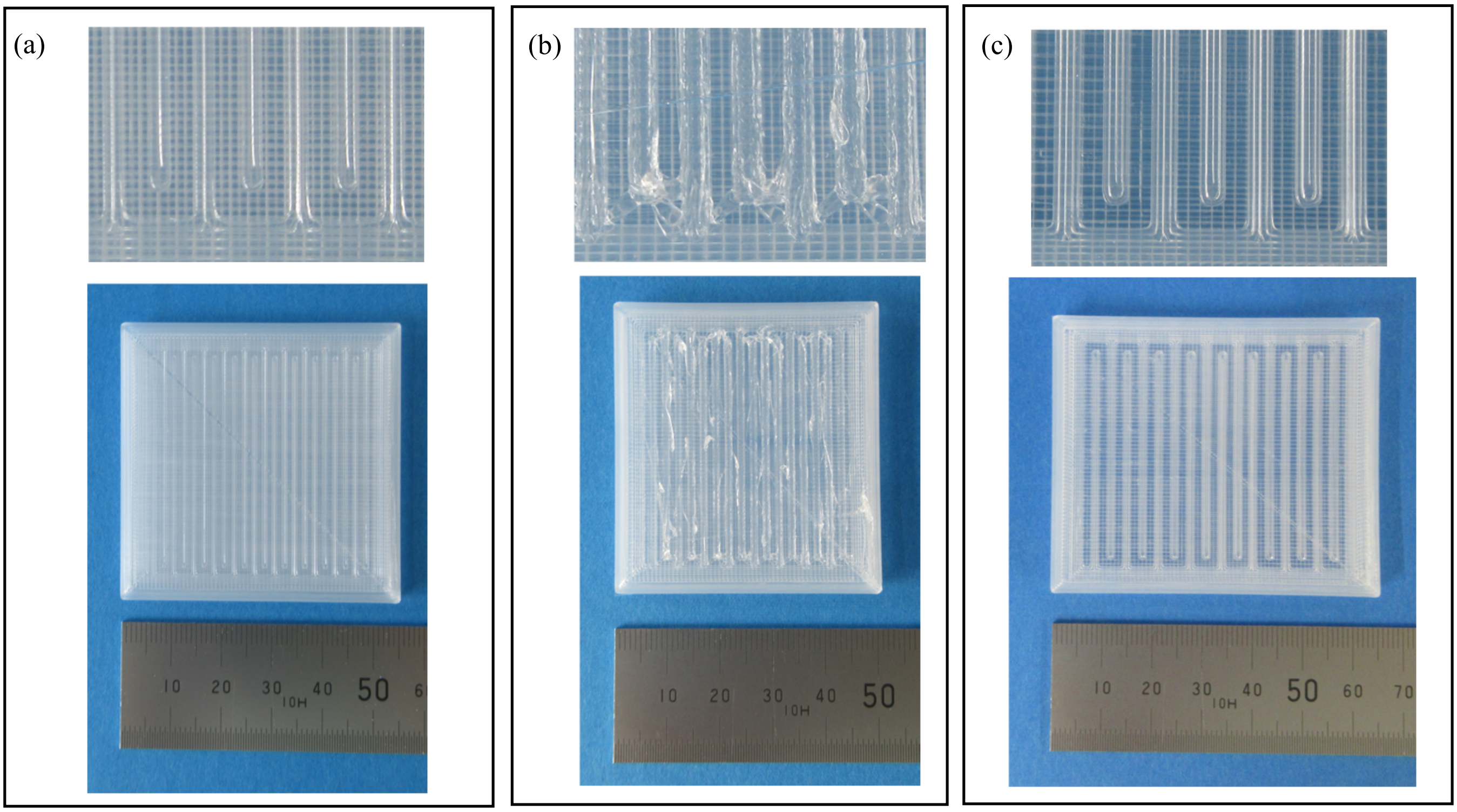

Fig. S8 Printing results for wall thicknesses of (a) 0.8 mm, (b) 1.2 mm, and (c) 1.6 mm.

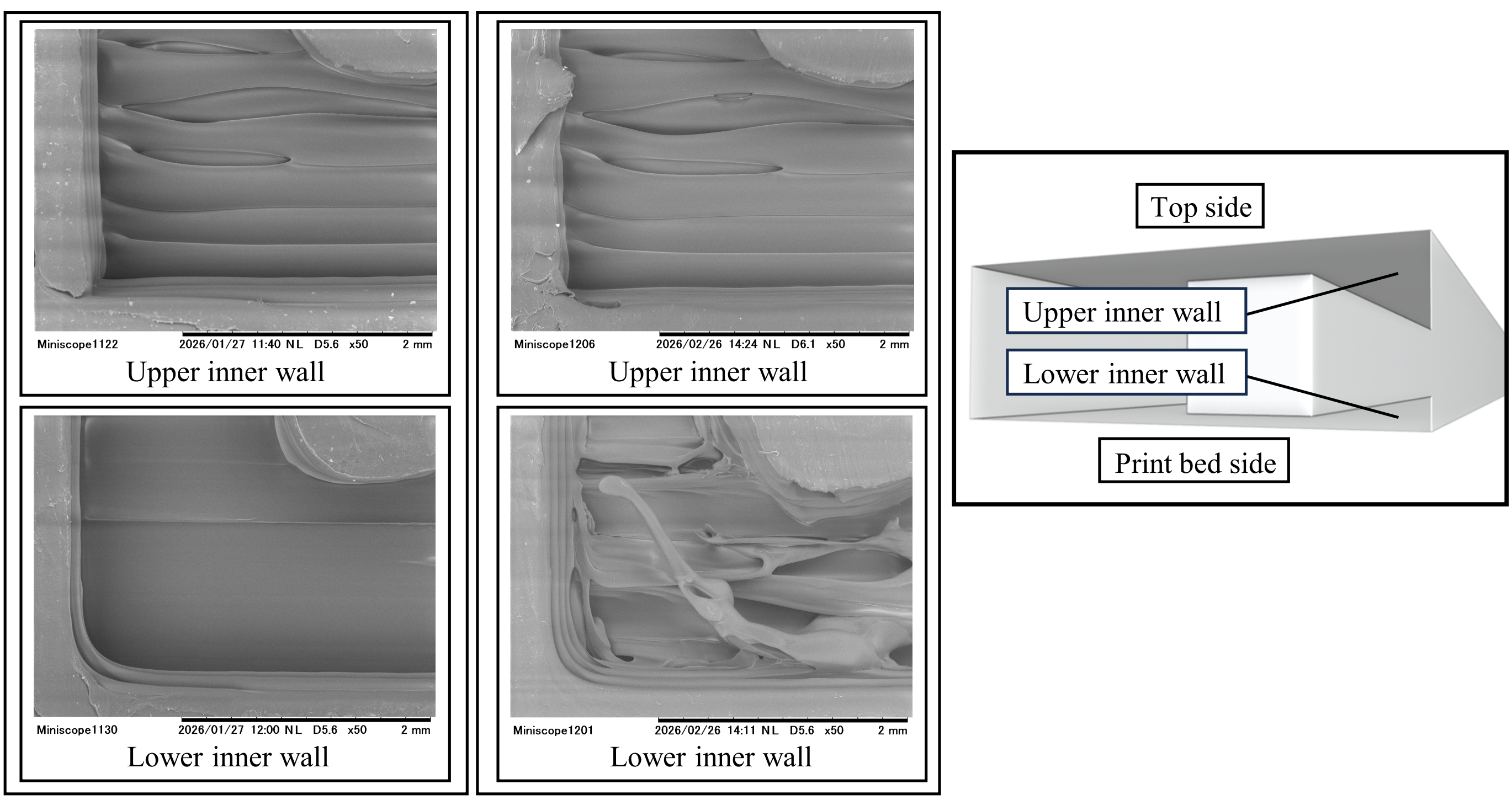

(a) large initial build height (approximately the thickness of three sheets of paper)

(b) small initial build height (approximately the thickness of one sheet of paper)

Fig. S9 Comparison of channel inner walls printed under different initial build height settings.

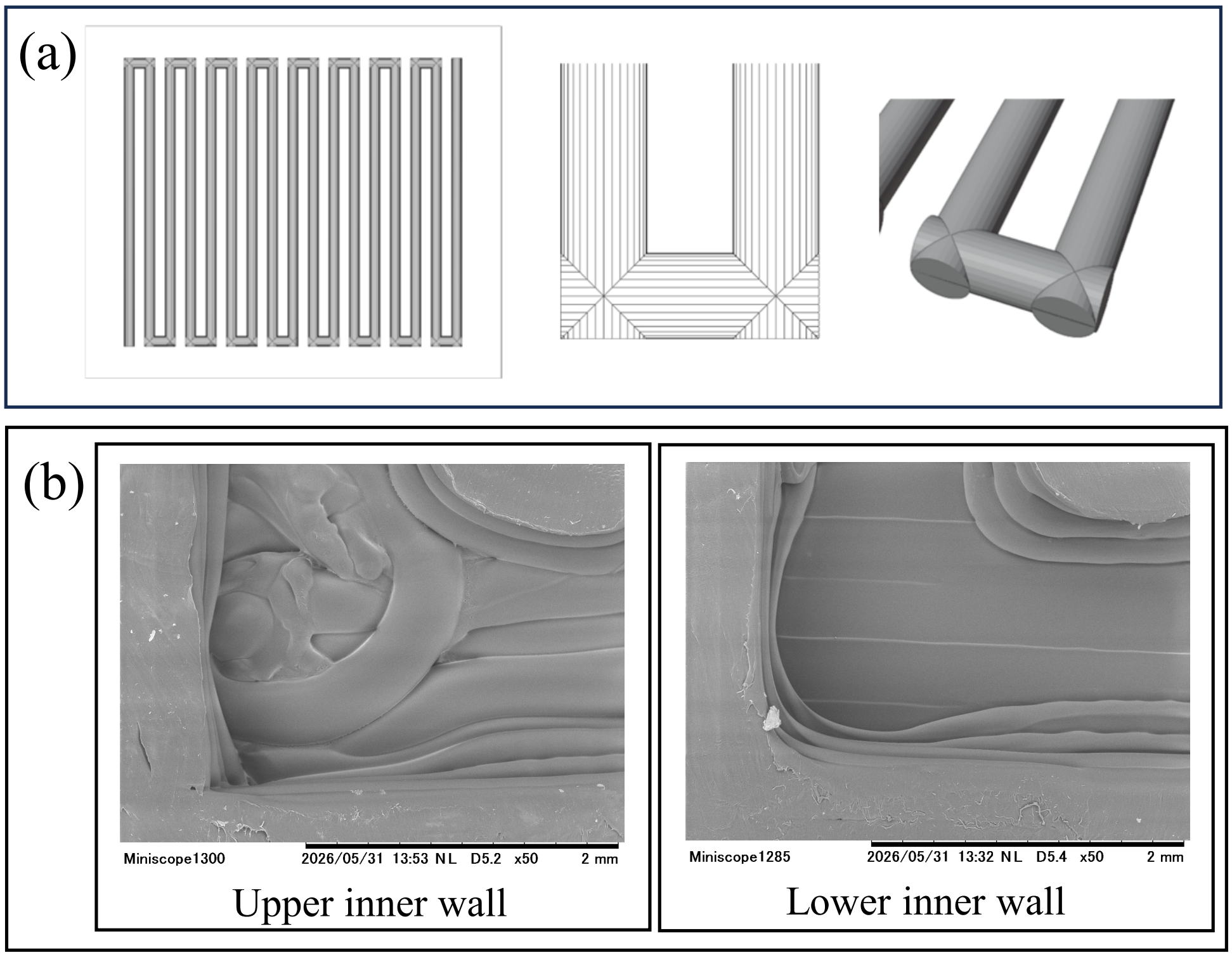

Unprocessed state

Fig. S10 (a) 3D models of the channel designed with a circular geometry. (b) SEM images of the channel designed with a circular geometry (unprocessed state).

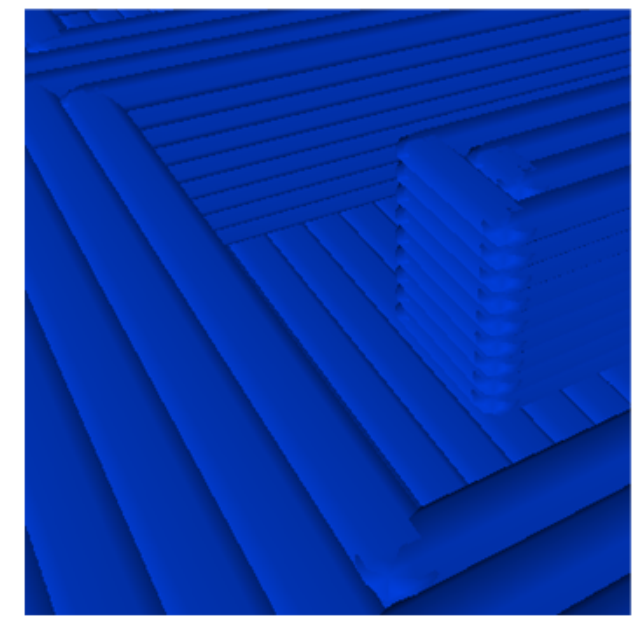

Fig. S11 Result of slicer software calculation of the internal channel structure of a fluidic chip (horizontal cross-sectional view).

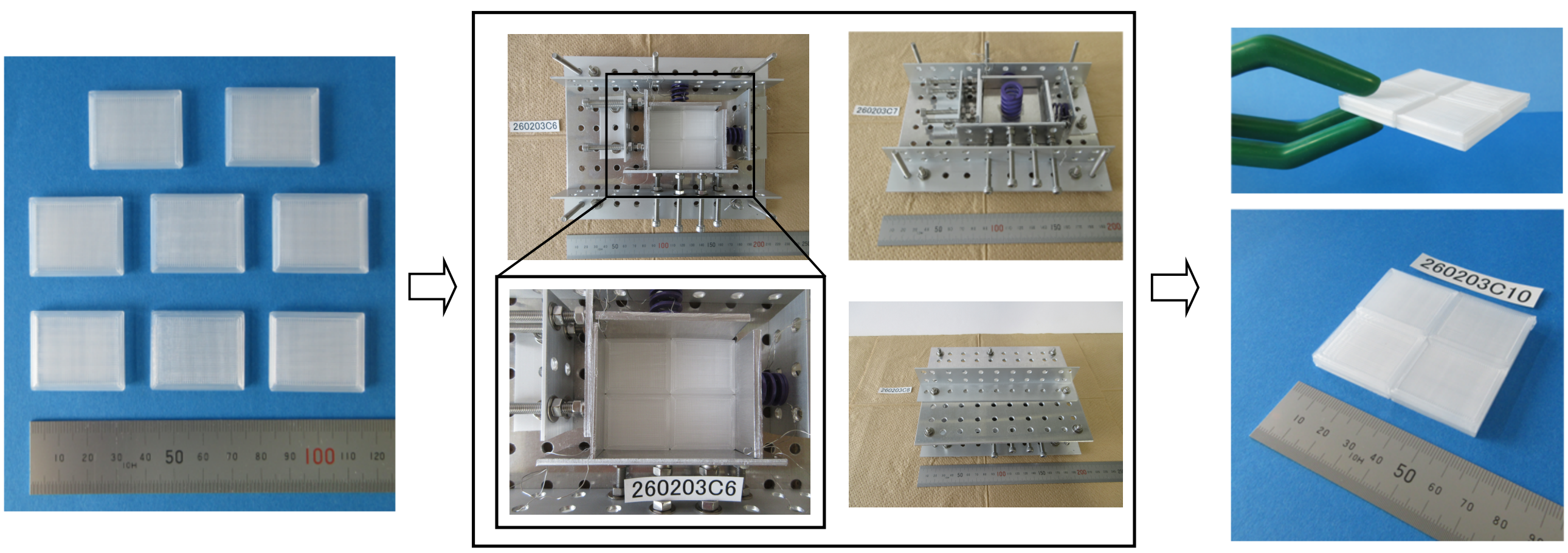

(a) Test pieces were printed.

(b) Test pieces were compressed and heated. (photograph taken before heating)

(c) Test pieces were lightly adhered.

Fig. S12 Schematic of the compression experiment.

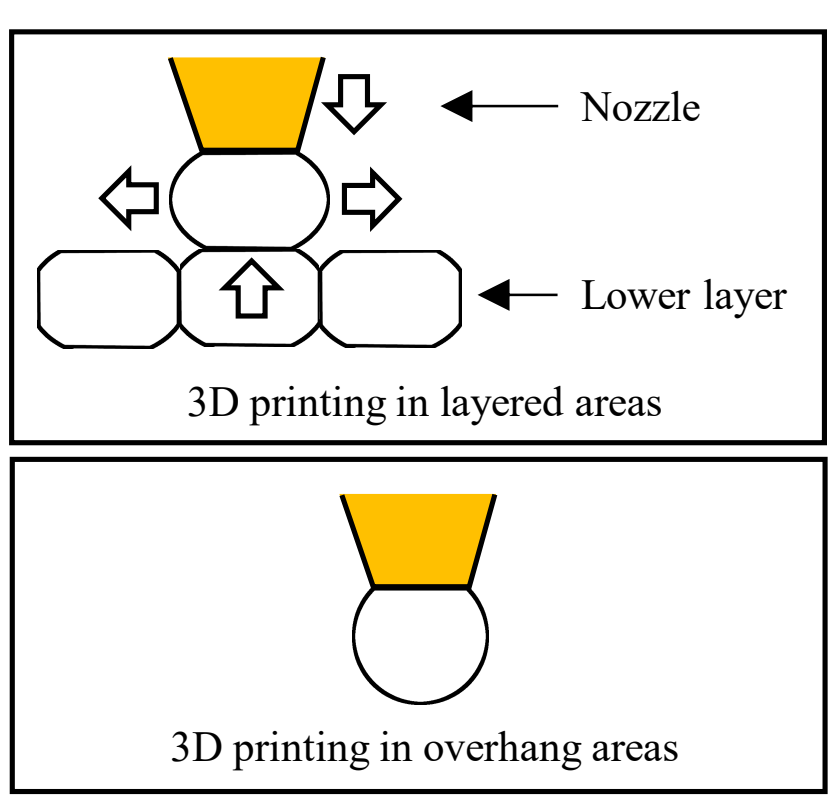

Fig. S13 Illustration explaining the speculated gap difference.

(a)

No compression process
No solvent process

Compression-processed only

Solvent-processed only

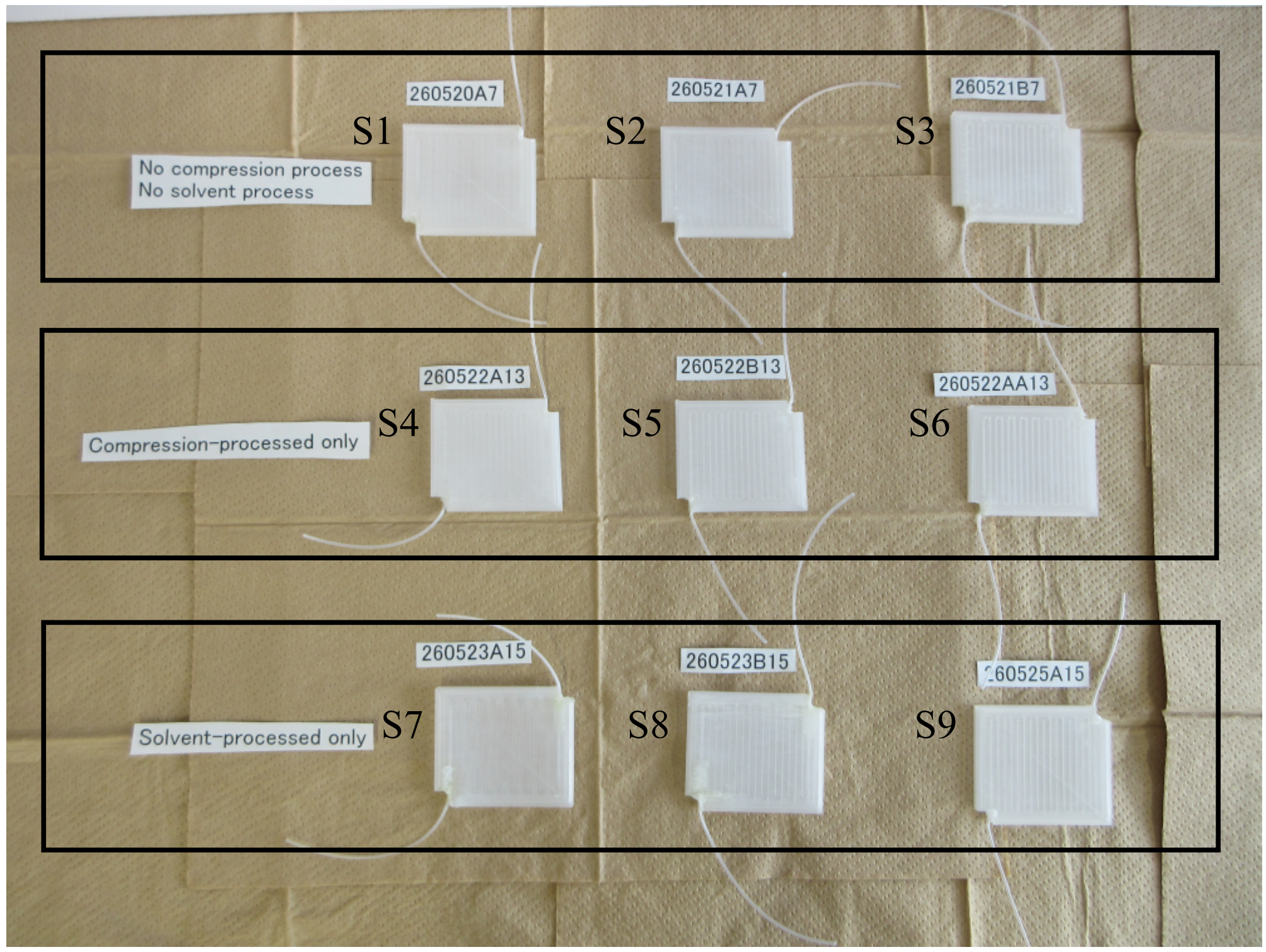

Polypropylene fluidic chips used for leakage testing

(b) Leakage-test results under static conditions

Water (S1-S9)	No leakage was observed.
EtOH (S1-S9)	No leakage was observed.

The solvent was filled into the internal channel, and the chip was left to stand for 5 minutes to check for leakage.

(c) Leakage-test results under flow conditions

Flow rate (ml/min)	0.5	1.0	2.0	3.0	5.0
Water (S1-S9)	No leakage was observed. (< 0.1 MPa)				
EtOH (S1-S9)	No leakage was observed. (< 0.1 MPa)				

The solvent was delivered using an HPLC pump, and each flow rate was maintained for 1 minute to confirm the absence of leakage. The pressure during the experiment was monitored by the HPLC pump. (< 0.1 MPa, corresponding to the minimum readable pressure of the HPLC pump, which displayed 0.0 MPa during the test)

Fig. S14 (a) Polypropylene fluidic chips used for leakage testing. Nine chips (S1–S9) were prepared under three processing conditions: No compression / No solvent (S1–S3), Compression only (S4–S6), and Solvent only (S7–S9). (b) Leakage-test results under static conditions. (c) Leakage-test results under flow conditions.

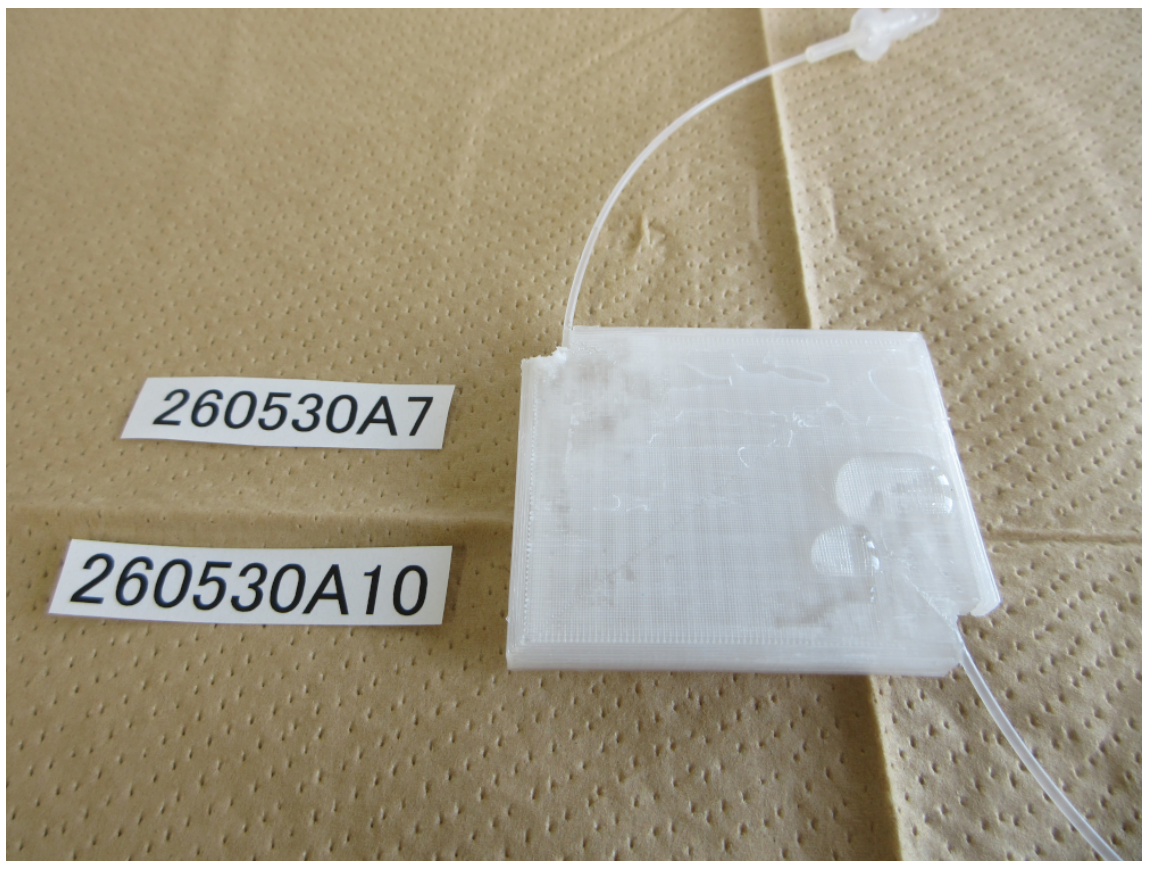

Fig. S15 Photograph of a PP chip that was 3D-printed with an extrusion multiplier of 0.9, showing leakage after water was injected into the channel (without compression or solvent-based post-processing).

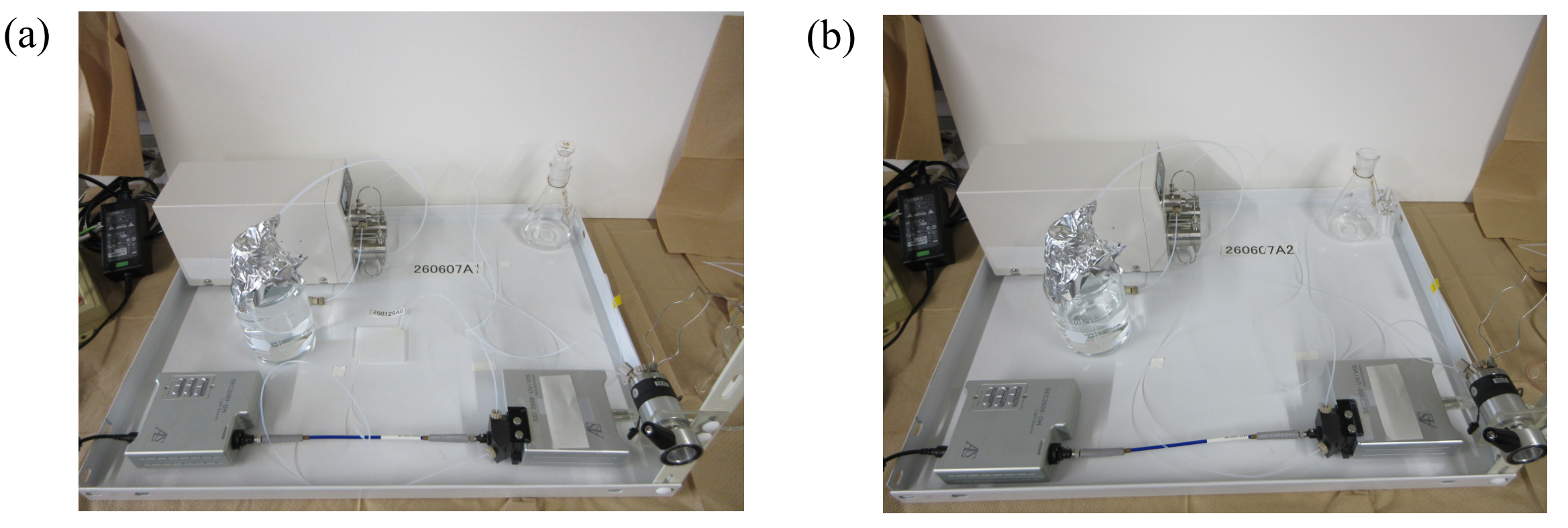

Fig. S16 (a) Photographs of the flow-injection analysis system using HPLC pump and the post-processed PP chip (a chip post-processed with approximately 20 mL of heptane was used). (b) Photographs of the flow-injection analysis system using HPLC pump without the PP fluidic chip.
(The flow cell used for this measurement is described in the Supporting Information of our previous report, *Analytical Sciences* **2022**, *38*, 583–589).

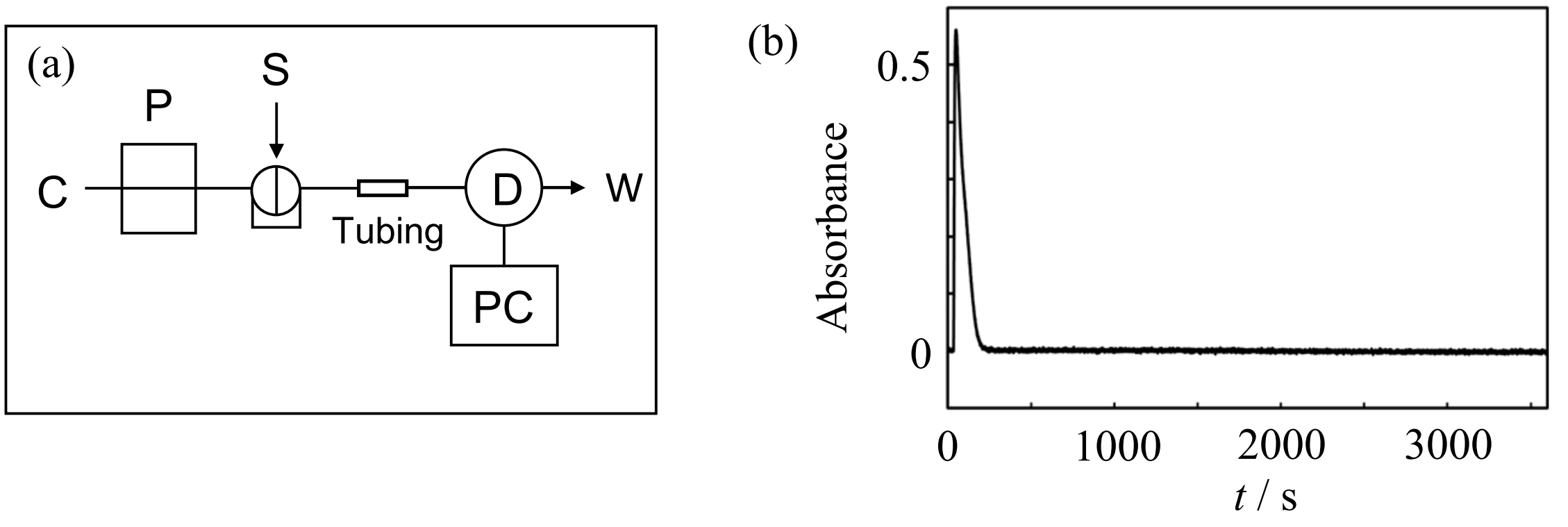

Fig. S17 (a) Schematic illustration of the flow-injection system using HPLC pump to measure the flow profile of Allura Red AC without the fluidic chip.: C, carrier solution (50% (v/v) ethanol–water solution); P, HPLC pump; S, sample injector; D, detector; PC, personal computer; W, waste; tubing was used as a connector. (b) Flow profiles of 5×10^{-4} M Allura Red AC measured without the fluidic chip.

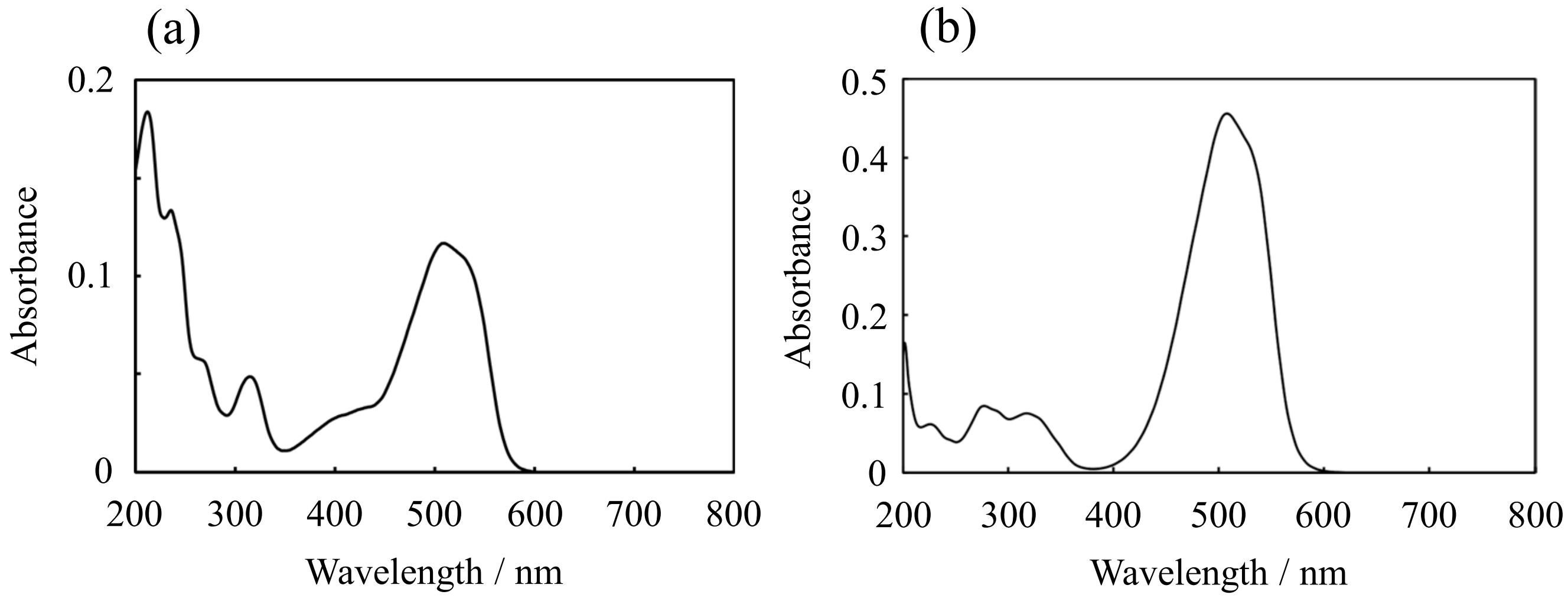

Fig. S18 (a) Absorption spectrum of 5 × 10^{-6} M Allura Red AC in 50% (v/v) ethanol–water solution. (b) Absorption spectrum of 1 × 10^{-5} M methyl orange in 0.1 M aqueous solution.

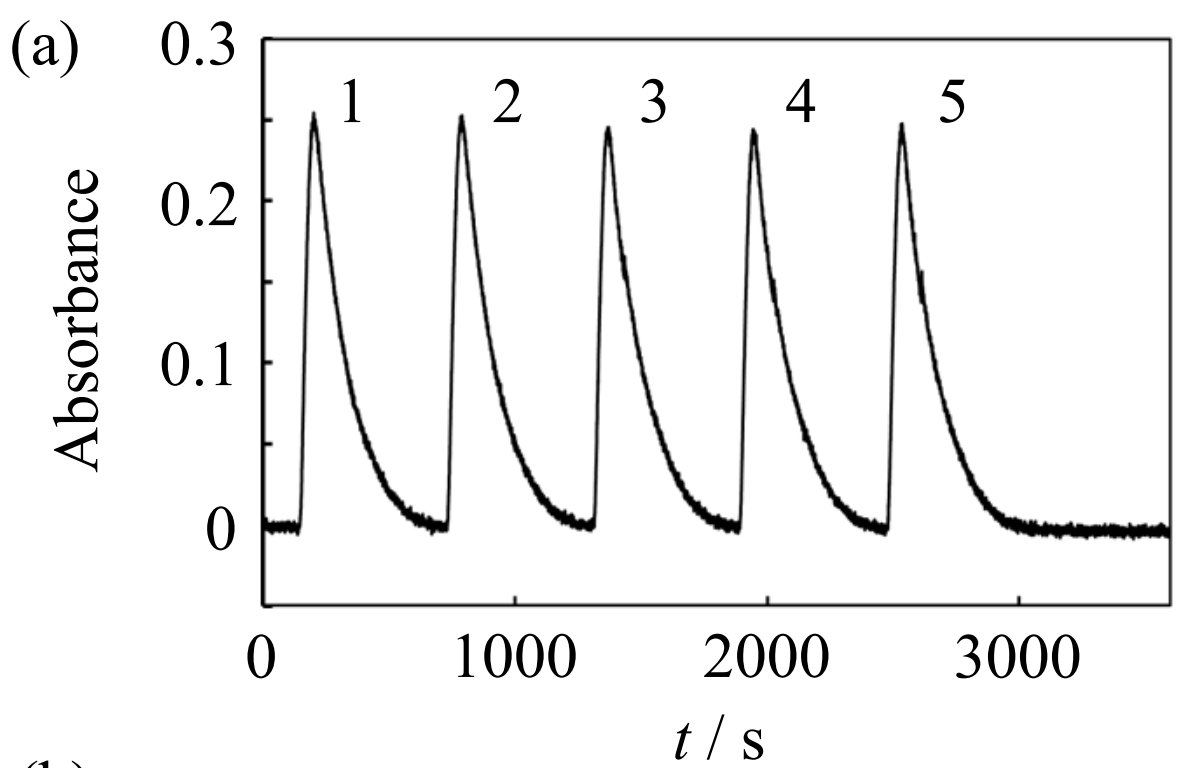

(b)

Calculation of Coefficient of variation (CV) from Figure S19a	Number	1	2	3	4	5	Average	CV(%)
	C / 10^{-4}M	4.98	5.03	4.95	4.90	4.91	4.954	1.07

Fig. S19 (a) Repeated injections of 5 × 10^{-4} M Allura Red AC. (b) Calculation of Coefficient of variation (CV) from Figure S19a

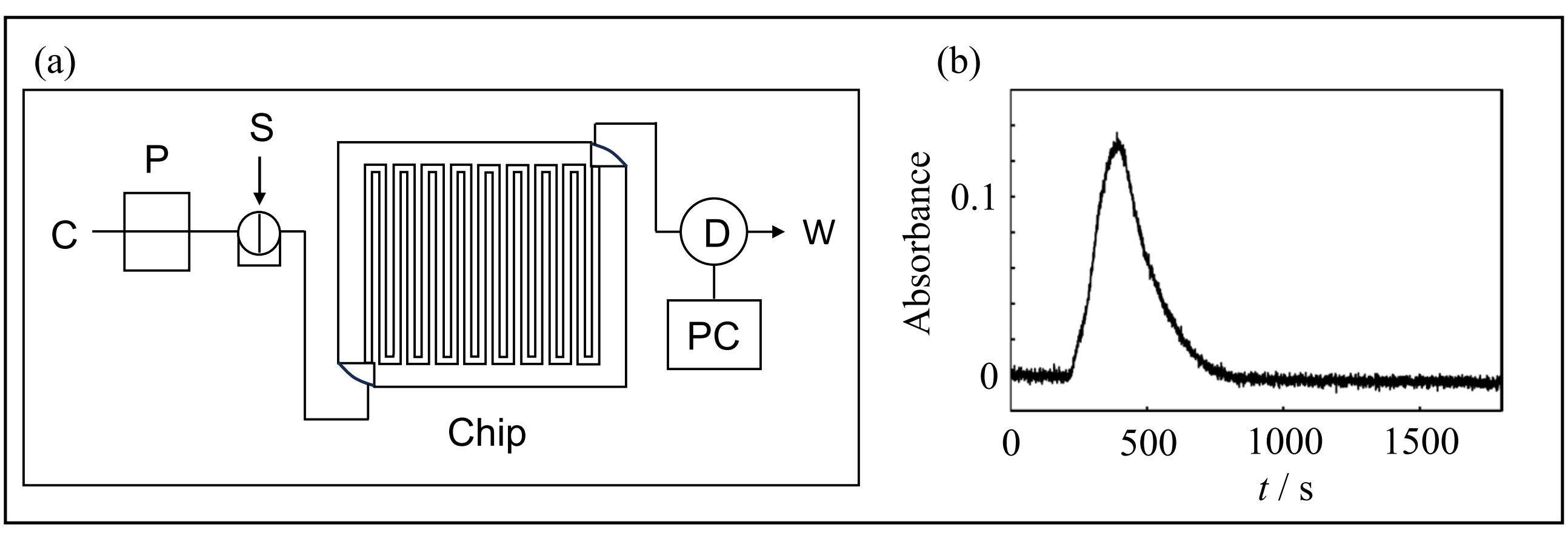

Fig. S20 (a) A schematic illustration of the flow injection system for measurement of methyl-orange: C, carrier solution (0.1 M hydrochloric acid aqueous solution); P, syringe pump; S, sample injector; D, detector; PC, personal computer; W, waste. (b) The flow profiles of methyl orange using a chip after post-processing with approximately 20 mL of heptane.

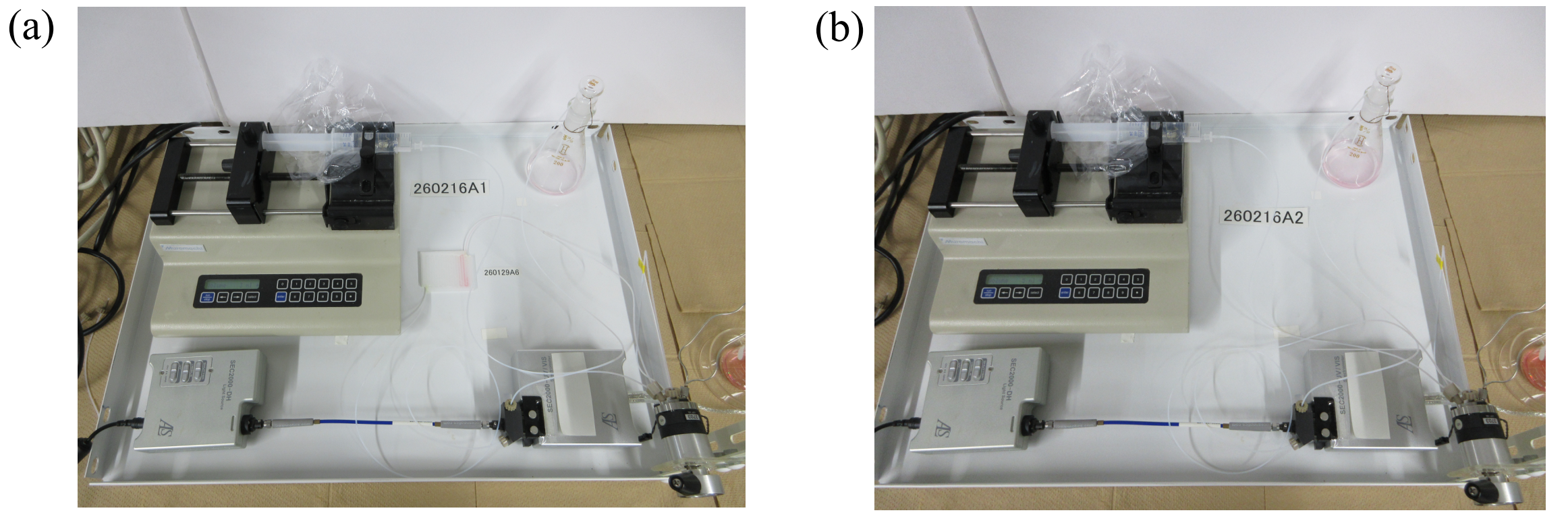

Fig. S21 (a) Photographs of the flow-injection analysis system using syringe pump and the post-processed PP chip (a chip post-processed with approximately 20 mL of heptane was used). (b) Photographs of the flow-injection analysis system using syringe pump without the PP fluidic chip.
(The flow cell used for this measurement is described in the Supporting Information of our previous report, *Analytical Sciences* **2022**, *38*, 583–589).

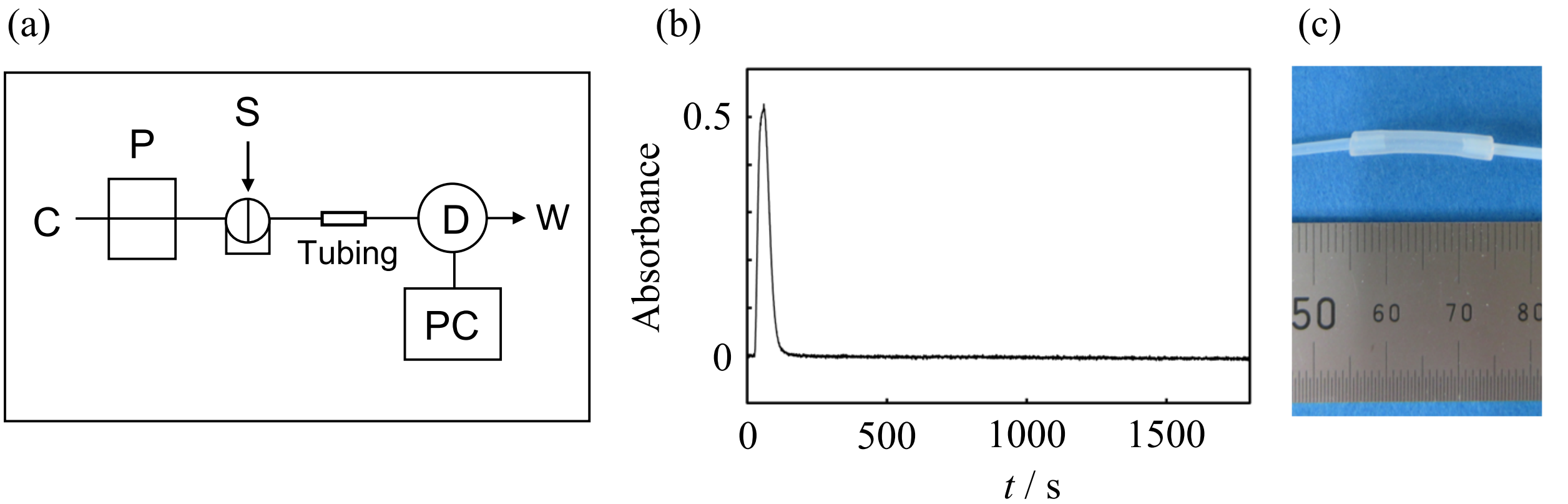

Fig. S22 (a) Schematic illustration of the flow-injection system using syringe pump to measure the flow profile of methyl orange without the fluidic chip.: C, carrier solution (0.1 M aqueous hydrochloric acid); P, syringe pump; S, sample injector; D, detector; PC, personal computer; W, waste; tubing was used as a connector. (b) Flow profiles of 1×10^{-4} M methyl orange in 1×10^{-3} M NaOH measured without the fluidic chip. (c) Example photograph of the tubing (silicone tube) used for connecting the flow lines.
